# Abnormal generation of IL-17A represses tumor infiltration of stem-like exhausted CD8^+^ T cells to demote the antitumor immunity

**DOI:** 10.1186/s12916-023-03026-y

**Published:** 2023-08-21

**Authors:** Ruochan Zhang, Kun Chen, Caifeng Gong, Zhiyuan Wu, Chungui Xu, Xing-Ning Li, Fei Zhao, Dongmei Wang, Jianqiang Cai, Aiping Zhou, Chunfeng Qu

**Affiliations:** 1 State Key Lab of Molecular Oncology, National Cancer Center/National Clinical Research Center for Cancer/Cancer Hospital, Chinese Academy of Medical Sciences and Peking Union Medical College, Beijing, 100021 China; 2 Immunology Department, National Cancer Center/National Clinical Research Center for Cancer/Cancer Hospital, Chinese Academy of Medical Sciences and Peking Union Medical College, Beijing, 100021 China; 3 Department of Medical Oncology, National Cancer Center/National Clinical Research Center for Cancer/Cancer Hospital, Chinese Academy of Medical Sciences and Peking Union Medical College, Beijing, 100021 China; 4 Department of Hepatobiliary Surgery, National Cancer Center/National Clinical Research Center for Cancer/Cancer Hospital, Chinese Academy of Medical Sciences and Peking Union Medical College, Beijing, 100021 China

**Keywords:** Stem-like exhausted CD8^+^ T cells, Interleukin 17A, ICB-immunotherapy, Tumor vascular endothelium, Anti-cancer therapy-related colitis

## Abstract

**Background:**

Variated anti-cancer therapies are combined with immune checkpoint blockades (ICBs) for improving ICB therapeutic efficacy. Occurrence of tissue damage is common that triggers multiple inflammatory cytokine generation. Gastrointestinal organs are the commonly affected. We investigated the impact of acute colitis on tumor infiltration of antigen-specific CD8^+^ cytotoxic T lymphocytes (CTLs) for controlling tumor growth and responding to antibody against PD-1 (anti-PD-1).

**Methods:**

Several tumor cell lines were inoculated into syngeneic mice subcutaneously or intra-hepatically. When tumor mass formed, activated CTLs were intravenously transferred into the tumor-bearing mice, that were given the drinking water containing 2% dextran sulfate sodium (DSS) for acute colitis induction. Tumor growth, infiltration of two exhausted CTL subsets, and the CTL interaction with tumor vascular endothelium were examined.

**Results:**

Acute colitis dampened CTL-mediated antitumor effects, correlating with IL-17A elevation in the inflamed intestine. In the tumor bed, stem-like exhausted CTLs, which were defined as PD-1^+^Slamf6^+^Tim3^−^, expressed higher IL-17A receptor heterodimers and lower leukocyte function-associated antigen-1 (LFA-1) than terminally exhausted CTLs did, that were defined as PD-1^+^Slamf6^−^Tim3^+^. IL-17A stimulation reduced LFA-1 surface expression on stem-like exhausted CTLs and the counterpart ICAM-1 (intracellular adhesion molecule-1) on tumor vascular endothelium. IL-17A stimulation suppressed the extravasation across tumor vascular endothelium and self-renewal of stem-like, not the terminally exhausted CTLs. Administration of anti-IL-17A neutralizing antibody to the colitis mice restored the CTL tumor infiltration and enhanced anti-PD-1 treatment efficacy against tumors. In 33 hepatocellular carcinoma patients being treated with anti-PD-1 plus antibody against vascular endothelial growth factor, disease progression of 15 patients, that exhibited serum IL-17A increase 24 h post-therapy as compared to pre-therapy level, was poorer than that of 18 patients that exhibited serum IL-17A no-increase.

**Conclusions:**

Abnormal generation of IL-17A mainly repressed tumor infiltration of stem-like exhausted CTLs. ICB-based immunotherapeutic efficacy could be upgraded with administration of anti-IL-17A, when treatment-related IL-17A elevation occurred due to tissue damage, such as acute colitis.

**Supplementary Information:**

The online version contains supplementary material available at 10.1186/s12916-023-03026-y.

## Background

Immunotherapy with immune checkpoint blockades (ICBs) which target the programmed death-1 (PD-1), or PD-1 ligand (PD-L1), or cytotoxic T-lymphocyte-associated protein-4 (CTLA-4) has revolutionized the management of multiple cancers. Due to non-response or resistance to mono-therapy in some patients, ICB combinations with other anti-cancer therapies are developed, including chemotherapy, radiation therapy, and targeted therapy. While patients benefit from the combined ICB immunotherapies, adverse event rates increased [1]. The damage can be caused by immunotoxicity, and also by chemotherapeutic regimens or irradiation. Gastrointestinal organs are involved commonly, with high incidence of gastrointestinal toxicities including diarrhea and mucositis [2]. Generally, interventional treatment was not recommended when low grade of adverse events occurred [3]. It is uncertain whether ICB-mediated anticancer efficacy is compromised in the patients experiencing acute damage of mucosal barrier, such as acute colitis.

Interleukin 17A (IL-17A) and Th17 cells critically participate in the pathogenesis of gut inflammation [4, 5]. Generally, IL-17A is expressed in the barrier surface tissues to maintain a healthy microbial population for protection. When the tissue barrier is damaged, abnormally generated IL-17A modulates and amplifies signals locally in a context-dependent manner in the pathogenesis of diseases and promotes disease progression through IL-17A receptor (IL-17R) heterodimers, IL-17RA, and IL-17RC. IL-17R is expressed ubiquitously and non-hematopoietic cells are generally the primary responders to IL-17. IL-17R expression was also identified in the immune cells [5, 6]. In cancers, abnormally generated IL-17A through the IL-17R signaling in multiple types of cells promotes disease development. It was demonstrated that IL-17A activates certain immune cell types, such as myeloid-derived suppressor cells (MDSCs) and neutrophils to suppress antitumor immunity and promote cancer development [5, 7–10]. IL-17A also promote tumor angiogenesis independent of the conventional vascular endothelial growth factor (VEGF) [5, 7, 11–14]. Elevation of IL-17A level was observed in some ICB-treated patients with immune-related adverse events [3, 15], and also in the patients treated with irradiation or chemotherapeutic agents [16, 17]. Compelling evidences demonstrate that the ICB immunotherapeutic efficacy depends significantly on the intra-tumoral, stromal, or invasive marginal CD8^+^ cytotoxic T lymphocytes (CTLs) but not the circulating CD8^+^ T cells [18]. IL-17A acts directly on CTLs in tumor microenvironment remains ambiguous.

Both in cancers and in chronic viral infections, two subsets of PD-1^+^ CTLs were identified that displayed distinct responses to anti-PD-1/PD-L1 [19–22]. Phenotypically, “terminally exhausted” CTL subsets express high levels of PD-1 and Tim3. The subset of “stem-like exhausted” CTLs express Slamf6 or CXCR5, and PD-1 at an intermediate level, but no Tim3. Functionally, “stem-like exhausted” CTLs expand vigorously upon inhibitory receptor blockade or specific antigen stimulation, exhibiting features of central memory and exhausted T cells. The “stem-like” subset persists long term and can differentiate into the subset of “terminally exhausted” CTLs which are prone to undergo apoptosis. Transcription factor Tcf1, encoded by the *Tcf7* gene, is essential for the stem-like functions of these cells. The “stem-like exhausted” CTL subset confers the capacity for controlling tumor growth in response to ICBs and vaccination [21, 22]. To kill the tumor cells, CTLs require to transmigrate across the tumor vasculature from bloods. Tumor blood vessels are abnormal, both structurally and functionally relative to those of nonmalignant tissues, restraining the effector T cell infiltration [23, 24]. Anti-angiogenesis by inhibiting proangiogenic signaling was combined with ICBs for enhancing antitumor immunity [1, 23, 25]. ICB-immunotherapy efficacy relies on the presence of intra-tumoral “stem-like exhausted” CTLs rather than on the lone reversal of T cell exhaustion programs [19, 21, 22]. To achieve better ICB-immunotherapeutic efficacy, it is required to understand the impacts of intestinal inflammation-related IL-17A generation on the tumor infiltration of two CTL subsets.

## Methods

### Cell lines and reagents

Murine melanoma B16F10 and B16-OVA, and human umbilical vein endothelial cells (HUVECs) were purchased from the National Infrastructure of Cell Line Resource (Beijing, China). Murine hepatoma Hepa1-6, colon carcinoma CT26.CL25, endothelial cell C166, and human hepatocellular carcinoma (HCC) cell lines (HepG2, Hep3B) were purchased from the American Type Culture Collection (ATCC). We transfected Hepa1-6 with a pcDNA3.1-OVA [26] to generate Hepa1-6-OVA. Cells were cultured following the suppliers’ instructions.

The information of used reagents including antibodies and primers is listed in Additional file 1: Table S1, Table S2.

### Mice and tumor models

C57BL/6 mice and BALB/c mice were purchased from Beijing HFK Bioscience Company. C57BL/6 background CD45.1 mice, OT-I mice (OVA_257–264_ peptide-specific CD8 TCR-transgene), and Pmel-1 mice (gp100_25–33_ peptide-specific CD8 TCR-transgene) were purchased from Aniphe Biolaboratory (Jiangsu, China). Study protocols (NCC2021A037) involving mice were approved by the Institutional Animal Care and Use Committee of the National Cancer Center/Cancer Hospital, Chinese Academy of Medical Sciences (NCC/CH, CAMS).

In each female C57BL/6 mouse, 5 × 10^5^ B16-OVA or B16F10, or 3 × 10^6^ Hepa1-6-OVA cells in 100-μl PBS were injected subcutaneously. In some experiments, B16-OVA cells in 20-μl PBS were inoculated into a mouse liver. Five days after tumor cell inoculation (D5), each mouse received 1 × 10^6^ activated peptide-specific CD8^+^ T cells via tail vein injection. Before cell transfer, CD8^**+**^ T cells were purified from splenocytes of OT-I mice or Pmel-1 mice using a mouse CD8^**+**^ T cell Isolation Kit (Miltenyi, Germany) with > 95% purity and stimulated with 200 ng/ml of OVA_257–264_ or gp100_25–33_ peptides for 4 days. After cell transfer, acute colitis was induced in some mice by providing drinking water containing 2% dextran-sulfate-sodium (DSS, 36–50 kDa) between D5 and D12 as reported [27]. In some experiments, 4 doses of neutralizing anti-IL-17A antibody (αIL-17A, Clone#17F3) were injected intraperitoneally into the mice that were given DSS drinking water. To test the combined effect of anti-PD-1 (αPD-1, Clone# 29F.1A12) and αIL-17A, BALB/c mice were inoculated with 2.5 × 10^5^ CT26.CL25 cells subcutaneously, given 2% DSS drinking water from D5 to D12. One group of the mice received 4 doses of αIL-17A, one group received 4 doses of αPD-1, one group received 4 doses of αIL-17A plus 4 doses of αPD-1, and one group received isotype IgG. Each dose contained 150-μg protein. Tumor growth was measured every 2–3 days and tumor volume was calculated as (width^2^ × length × 0.5).

### Flow cytometry (FCM)

Tumor tissues were treated as we reported previously [26]. FCM analysis was performed using standard laboratory protocols. Data were acquired in LSR-II (BD Biosciences, USA) and analyzed using Flowjo software (Tree Star, USA).

### CD8^+^ T cell proliferation, adhesion, and trans-endothelium migration

CD8^**+**^ T cells were isolated from OT-I mice, stimulated with 200 ng/ml of OVA_257–264_ peptides in the presence of 0, 2, and 20 ng/ml of recombinant mouse IL-17A (rmIL-17A, Peprotech, USA). Cell numbers were counted at different time points. In some experiments, the purified CD8^**+**^ T cells were labeled with 2.5 μM CFSE (eBioscience, USA) before stimulation.

CD8^**+**^ T cell transmigration across vascular endothelium was assayed according to the literature [28]. C166 cells were pre-treated with B16-OVA cell medium (B16-OVA/CM) with or without a supplement of 20 ng/ml of rmIL-17A for 48 h. Transwell inserts (5 μm in pore size) were coated with 0.1% gelatin, 7.5 × 10^3^ C166 cells were seeded onto the upper surface of each insert and cultured overnight to form confluent monolayers. After removal of the medium, 3 × 10^5^ activated CD8^**+**^ T cells were added into each upper chamber and continued to culture for 4 h. The cells migrated into the lower chamber were collected, counted, and analyzed with FCM. In some experiments, 2 μg/ml of neutralizing antibody against IL-17RA, or IL17RC (R&D, USA) was included in the medium.

CD8^**+**^ T cell adhesion to vascular endothelium was analyzed according to the report [29]. In each well of 6-well plates, 1 × 10^5^ C166 cells were seeded and treated with B16-OVA/CM with or without a supplement of 20 ng/ml of rmIL-17A for 48 h. Activated CD8^**+**^ T cells (2 × 10^5^ cells/well) were added for another 16 h. Nonattached CD8^**+**^ T cells were washed out using 37ºC pre-warmed PBS three times. All cells were then recovered with trypsin, and CD8^**+**^ T cells adhered to C166 cells were analyzed with FCM.

### Multiplex immunohistochemistry (mIHC)

Opal Multiplex IHC assay kit (PerkinElmer, USA) was used for mIHC staining according to the literature [30]. Slides were scanned with a Pannoramic MIDI slide scanner (3DHISTECH, Hungary) at 20 × magnification and analyzed with HALO image analysis platform (Indica, USA). The relationship of CD8^+^ T cells with tumor vasculature was analyzed as reported [31].

### Determination of nitric oxide (NO) and endothelial NO synthase (eNOS)

In 6-well plates, 1.5 × 10^4^ C166 cells/well were treated with B16-OVA/CM with or without a supplement of 20 ng/ml of rmIL-17A. NO assay kit (Beyotime, China) was used to determine NO generation following the manufacturer’s specifications. Immunoblot was performed to determine the expression levels of total eNOS, phosphorylated-eNOS at Ser1177.

### Patients

Fifty patients with advanced HCC were treated with anti-PD-1 (sintilimab) plus anti-VEGF (IBI305) every 3 weeks in a phase Ib clinical study conducted at NCC/CH. The therapeutic efficacy was reported previously [32]. From 33 patients, we obtained the paired serum samples before therapy and 24 h post-therapy, and their demographics and baseline characteristics are listed in Additional file 1: Table S4. ProcartaPlex Human Cytokine/Chemokine/Growth Factor Panel (Affymetrix, USA) was used to determine serum levels of multiple cytokines, including IL-17A.

### Statistical analysis

Version 8.0 GraphPad Prism was used for statistical analysis. Continuous variables were compared with unpaired Student’s *t-*test between two groups, One-way ANOVA between more than two groups. Fisher’s exact test was used to compare categorical variables. Survival curves of treated patients were generated using the Kaplan–Meier method and compared with the log-rank test. *P-*value of less than 0.05 was considered to be statistically significant.

## Results

### Acute colitis dampened CTL-mediated antitumor effects

**Fig. 1:**
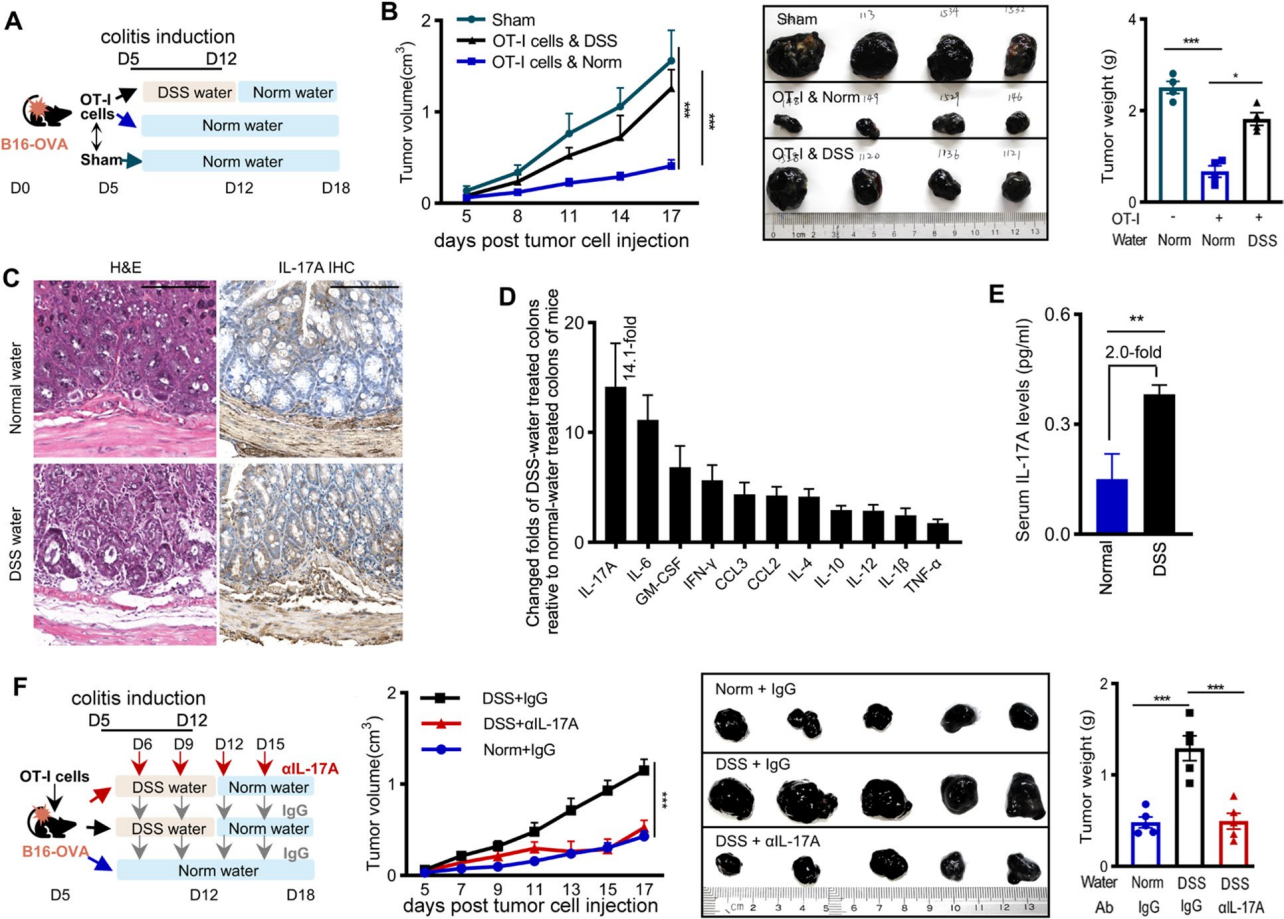
CTL-mediated antitumor effect in mice models with acute colitis and IL-17A generation. **A** Experimental scheme, all mice were sacrificed on D18 when tumors in sham-treated mice reached ≥ 2.0 cm in length. **B** Tumor growth curve, and the weight and images of tumors removed on D18, one of three independent experiments. **C** Representative histology (HE) and IL-17A immunochemistry staining of the mouse intestine detected on D18. Scale bars, 100 μm. **D** Folds of indicated cytokines in the colons of DSS-water-fed mice (*n* = 4) relative to the normal-water-fed mice (*n* = 4) determined on D18. **E** Serum IL-17A on D18 in the indicated group of mice. **F** Treatment of αIL-17A, each group included 5 mice and repeated twice. Tumor growth curve, and the weight and images of tumors removed on D18 in the indicated group, one of two independent experiments. Data in bar graphs are presented as mean ± SD, compared by two-tailed Student’s *t-*test (two groups), or One-way ANOVA with Tukey’s multiple comparisons (three groups). Each dot represents one mouse. *, *P* < 0.05; **, *P* < 0.01; ***, *P* < 0.001

To mimic an acute damage to gastrointestinal organs in humans, we induced an acute colitis in several tumor-bearing mice models by providing 2% DSS drinking water [27] (Fig. 1A). Tumor growth and tumor weight in the CTL-transferred mice, that received OT-I cells and fed normal drinking water consistently (OT-I & Norm), was significantly slower than that in the sham-treated mice, that received no OT-I cells and fed normal drinking water (Fig. 1B). These results confirmed the potent antitumor effects mediated by CTLs. However, tumor grew rapidly in the CTL-transferred mice when DSS water was given for one week (OT-I & DSS), exhibiting failure of tumor control (Fig. 1B). Tumor growth in mice that were inoculated subcutaneously with Hepa1-6-OVA and in those with B16-OVA cells in livers (Additional file 2: Fig. S1A, S1B) showed similar to that in the mice bearing B16-OVA subcutaneously.

To validate the finding with model OVA-engineered tumor cell lines, we inoculated C57BL/6 mice with B16F10 cells that constitutively express the tumor-associated antigen gp100. The mice received intravenously gp100-specific T cells on D5, treated as in the B16-OVA-bearing mice. Tumor growth in the DSS-water-fed mice was significantly faster than that in the normal-water-fed mice (Additional file 2: Fig. S1C). All these results indicated that CTL-mediated antitumor immunity was dampened when acute colitis occurred.

### Impairment of CTL-mediated antitumor effects was related to IL-17A elevation in the inflamed intestine

We collected the colons when mice were sacrificed on D18. The mice fed DSS water displayed colitis changes in morphology (named colitis mice) (Fig. 1C). We prepared the interstitial fluid of colon tissues to quantify the generation of multiple cytokines that represent Th1-, Th2- and Th17-related responses to the damage. All tested cytokines increased in the colons of colitis mice. Notably, compared with the normal-water-fed mice (named normal mice), IL-17A increased mostly (14-folds), followed by IL-6 and GM-CSF (Fig. 1D), which are all related to Th17 [5]. More IL-17A-producing cell numbers were detected in the colons of colitis mice, with about 3.2-folds of increase as a comparison to normal mice (Fig. 1C, Additional file 2: Fig. S2). CD3^+^CD4^+^ Th17 cells were identified as the major IL-17A-producing cells, followed by CD3^+^CD8^+^ Tc17 cells (Additional file 2: Fig. S2). Colitis mice also displayed IL-17A elevation in blood, but increased 2-folds only (Fig. 1E). When the colitis mice were treated with a neutralizing antibody against IL-17A (αIL-17A), tumor growth was significantly inhibited compared with the mice that were treated with isotype IgG (Fig. 1F). These data indicated that colitis could dampen the CTL-mediated antitumor effects, relating to elevated generation of IL-17A in inflamed intestine.

### Acute colitis-induced IL-17A reduced the CD8^+^ T cells in tumor tissues

**Fig. 2:**
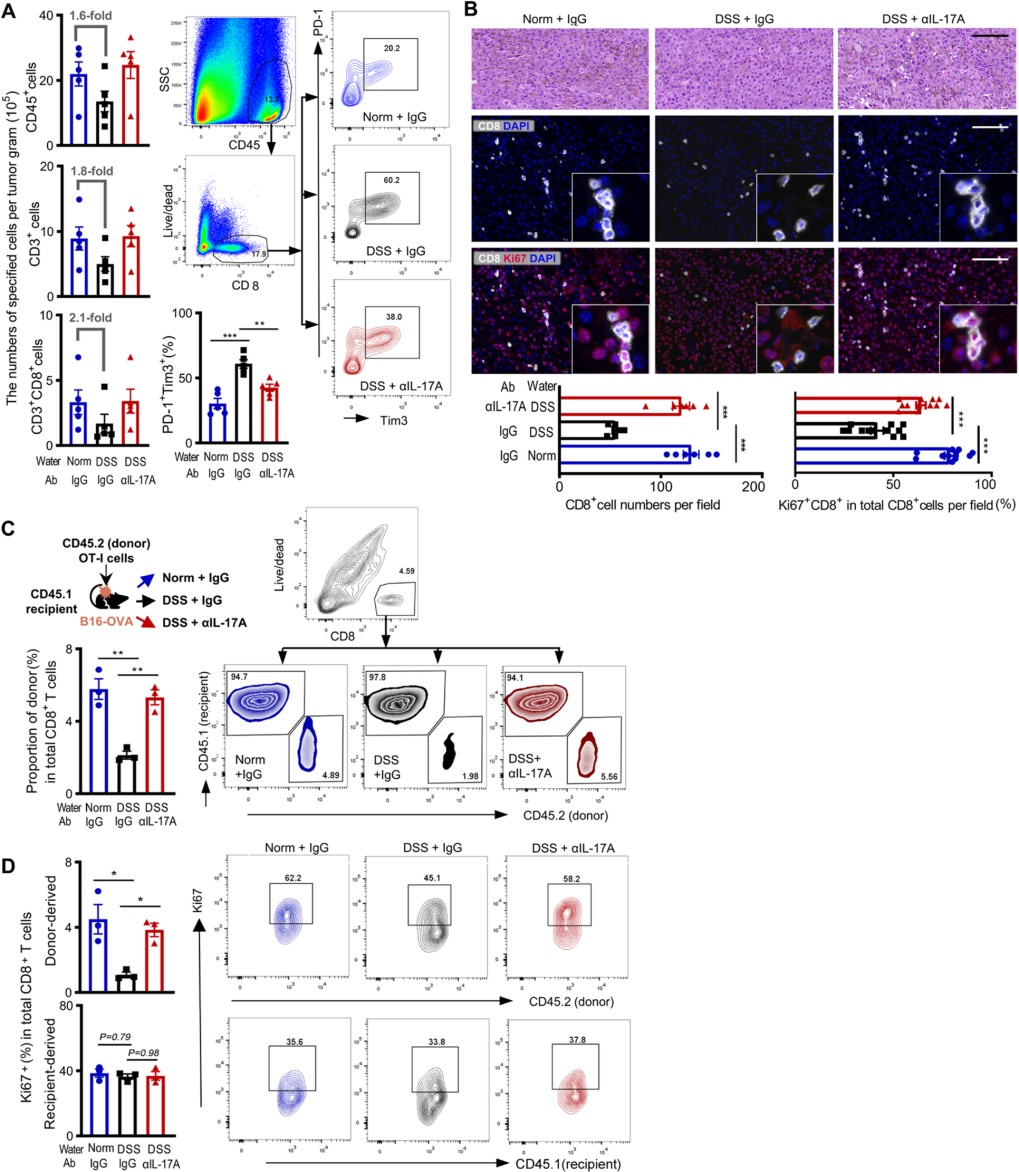
Immune cell infiltration in the tumor bed. **A** Numbers of CD45^+^ total immune cells, CD3^+^ T cells, CD3^+^CD8^+^ T cells, and PD-1^+^Tim3^+^ cell percentage of total CD8^+^ T cells in the tumors of differently treated mice. FCM plots show one representative of five independent experiments. **B** Representative images of HE staining, CD8 (white), and Ki67 (red) staining of B16-OVA tumors as indicated. Nuclear was stained with DAPI (blue). Scale bars, 100 μm. **C**, **D** Each CD45.1 mouse was injected subcutaneously with B16-OVA cells, received CD45.2 OT-I cells via tail vein injection, and treated as did in Fig. 1F (*n* = 3 per group). FCM plots show one representative of three independent experiments. Bar graphs show the proportion of donor-derived (CD45.2) cells in total tumor-infiltrating CD8^+^ T cells (**C**); the percentages of the donor-derived Ki67^+^CD8^+^ (upper) and recipient-derived Ki67^+^CD8^+^ (low) T cells in total tumor-infiltrating CD8^+^ T cells (**D**). Data in bar graphs dare presented as mean ± SD and compared by One-way ANOVA. Each dot represents one mouse. *, *P* < 0.05; **, *P* < 0.01; ***, *P* < 0.001

In tumor tissues of colitis mice (DSS + IgG), the numbers of total immune cells (CD45^+^), CD3^+^ T cells, and CD3^+^CD8^+^ T cells reduced by 1.6-, 1.8-, and 2.1-folds, respectively, compared with that of the normal mice (Norm + IgG). More proportion of PD-1^+^Tim3^+^ exhausted CD8^+^ T cells presented in the tumors of colitis mice. When the colitis mice received αIL-17A (DSS + αIL-17A), infiltration of CD45^+^, CD3^+^, and CD3^+^CD8^+^ cells restored, while PD-1^+^Tim3^+^ exhausted CD8^+^ T cells reduced in the tumor tissues (Fig. 2A). Multiplex staining of tumor tissues from differently treated mice confirmed the CD8^+^ T cell reduction of colitis mice. Notably, the density of proliferative Ki67^+^CD8^+^ T cells reduced more profoundly in the tumor bed of colitis mice than in that of normal mice, associated with IL-17A elevation (Fig. 2B).

To understand the effects of colitis-related IL-17A on reducing CD8^+^ T cell numbers in the tumor bed, we transferred the activated OT-I cells from CD45.2 mice (donor) into B16-OVA-bearing CD45.1 mice (recipient) and treated the mice as did in Fig. 1F. The numbers of donor-derived CD8^+^ T cells (CD45.2^+^) in the tumors of colitis mice reduced more significantly (Fig. 2C). Particularly, the percentage of donor-derived proliferating CD8^+^T cells (Ki67^+^CD8^+^) decreased more profoundly in the tumors of colitis mice. While the colitis-induced IL-17A showed minor effects on the recipient-derived CD8^+^ T cells (Fig. 2D).

### Stem-like exhausted CTLs expressed higher IL-17R heterodimers

**Fig. 3:**
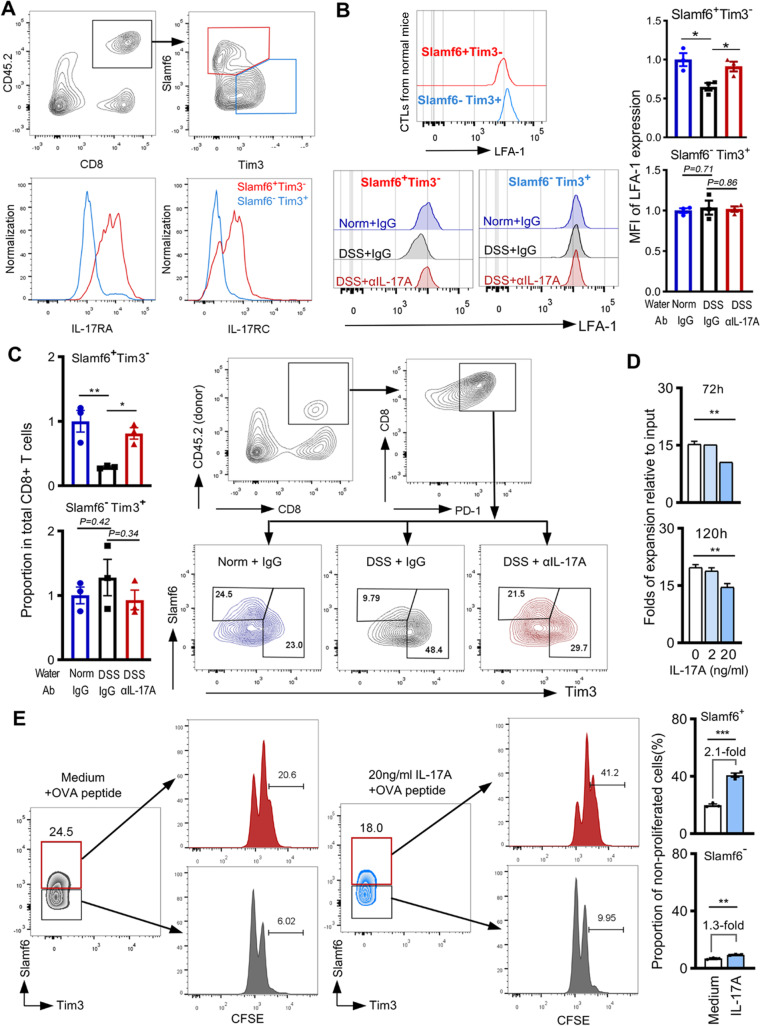
Expression of IL-17A receptor heterodimers and IL-17A effects on tumor infiltration and proliferation of two exhausted CD8^+^ T cell subsets. **A** Surface expression of IL-17RA and IL-17RC on two exhausted CTL subsets was examined in the B16-OVA tumor tissues of normal CD45.1 mice that received CD45.2 mouse OT-I cells. One representative of four independent experiments. **B** Surface expression of LFA-1 on two CTL subsets. The up one shows LFA-1 on specified cells isolated from normal mice tumors (one of two independent experiments); the low one shows those isolated from differently treated mice tumors (one of three independent experiments). Bar graphs show the average mean fluorescent intensity (MFI) of LFA-1. **C** Infiltration of two CTL subsets in the tumors of differently treated mice (bar graphs). FCM profiles show one representative of three independent experiments. **D** Purified OT-I cells were stimulated with 200 ng/ml of OVA_257–264_ peptides plus indicated concentration of rmIL-17A. Changed folds at the indicated time points relative to the baseline cell numbers. **E** Proliferation of CFSE-labeled OT-I cells after being stimulated for 48 with OVA_257–264_ peptides alone (medium) or supplement of 20 ng/ml of rmIL-17A. One representative of three independent experiments which are shown in Additional file 2: Fig. S6. Data in bar graphs are presented as mean ± SD and analyzed with Student’s *t-*test (two groups), One-way ANOVA (three groups). Each dot represents one independent repeat. *, *P* < 0.05; **, *P* < 0.01; ***, *P* < 0.001

IL-17 signals through the IL-17RA and IL-17RC [6]. We then examined the surface expression levels of IL-17RA and IL-17RC on two CTL subsets of tumor-infiltrated lymphocytes (TILs) based on the surface markers of Slamf6 and Tim3 [21]. The PD-1^+^Slamf6^+^Tim3^−^ stem-like CTLs were recognized to express higher levels of both IL-17RA and IL-17RC than the PD-1^+^Slamf6^−^Tim3^+^ terminally exhausted CTLs did (Fig. 3A). For validation, we retrieved two public data (GSE84105, GSE123235), that performed RNA-seq on two purified murine CTL subsets [19, 22], and confirmed the higher transcriptional levels of both IL-17RA and IL-17RC in the stem-like exhausted CTLs (Additional file 2: Fig. S3A, S3B). Transcription levels of IL-17RD, which serves as an alternate receptor subunit for IL-17A [33], showed no difference between the two CTL subsets (Additional file 2: Fig. S3C).

### Colitis-induced IL-17A suppressed the tumor infiltration and self-renewal of stem-like exhausted CTLs

Due to the significant reduction of effector CD8^+^ T cells in the tumor bed (Fig. 2C), we analyzed the surface expression of leukocyte function-associated antigen-1 (LFA-1) and very late antigen (VLA-4), which are typical integrins for T cell extravasation from blood into peripheral tissues [24]. Analysis of two public data (GSE84105, GSE123235) indicated that the “stem-like exhausted” CTLs expressed a lower level of both *Itgal* (*CD11a*) and *Itgb2* (*CD18*), two LFA-1 subunits, than the “terminally exhausted” CTL subset did (Additional file 2: Fig. S4A). Transcription of *Itgb1* (*CD29*), one VLA-4 subunit, displayed higher in the stem-like subset than in the terminal subset. However, *Itga4 (CD49d)*, another VLA-4 subunit, showed no difference in transcription between the two subsets (Additional file 2: Fig. S4B). We therefore mainly examined the LFA-1 expression on CD8^+^ T cell infiltration into the tumor bed of the current study. In the TILs of normal mice, the stem-like CTLs displayed lower surface expression of LFA-1 than the terminally exhausted CTLs did (Fig. 3B). Colitis-induced IL-17A profoundly reduced LFA-1 expression on the stem-like subset, but minorly affected LFA-1 on the terminally exhausted CTLs. After injection of αIL-17A into the colitis mice, LFA-1 surface expression was partially restored on the stem-like subset (Fig. 3B). Quantifying total CD8^+^ T cells in the tumors of colitis mice, the numbers of stem-like subset reduced significantly, associating with the IL-17A generation. No reduction of the terminally exhausted CTL numbers was observed (Fig. 3C). We used CXCR5^+^Tim3^−^ to recognize the stem-like subset [19] in some experiments and obtained the same results (Additional file 2: Fig. S5).

IL-17A on CTL proliferation was also examined by stimulating the OT-I cells with OVA_257–264_ peptides in the presence of rmIL-17A. Cell numbers reduced significantly after being treated for 72 h and 120 h when 20 ng/ml of rmIL-17A was included in the medium (Fig. 3D). Analysis of the CFSE-labeled OT-I cells showed that the IL-17A significantly suppressed the proliferation of PD-1^+^Slamf6^+^Tim3^−^ stem-like population but minorly inhibited the PD-1^+^Slamf6^−^ population (Fig. 3E, Additional file 2: Fig. S6). No difference in cell apoptosis, cytotoxicity on target tumor cells, and specified cytokines release were observed when IL-17A was included in the cultures (Additional file 2: Fig. S7).

### Colitis-induced IL-17A exaggerated tumor vascular endothelium dysfunction to reduce stem-like exhausted CTL extravasation

**Fig. 4:**
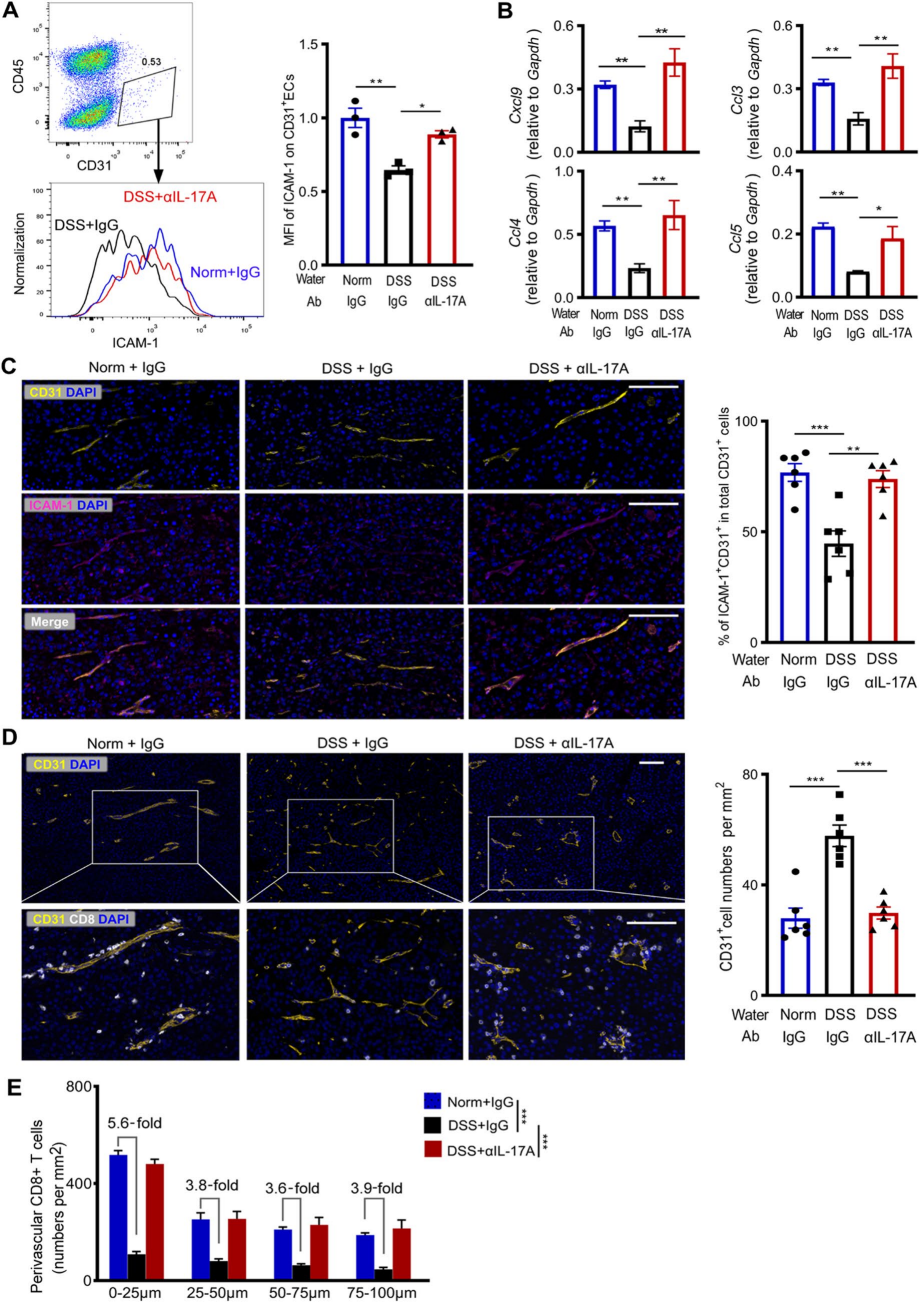
CTLs in relation to blood vessels in the tumor bed. **A** Surface ICAM-1 expression on tumor vascular endothelial cells (ECs) of differently treated mice, one representative of three independent experiments. Bar graph shows the ICAM-1 average mean fluorescent intensity (MFI) relative to normal mice. **B** *Cxcl9*, *Ccl3*, *Ccl4*, and *Ccl5* transcriptional levels in B16-OVA tumors of indicated mice. **C**, **D** Representative images of tumor tissues as indicated. Nuclear was stained with DAPI (blue). Scale bars, 100 μm.** C** Staining of CD31 (yellow) and ICAM-1 (magenta). Bar graph shows the percentage of ICAM-1 + CD31 + double-positive cells in total CD31 + vessels. **D** Staining of CD31 (yellow) and CD8 (white). Bar graph shows the average density of CD31 + vessels. **E** Perivascular CD8^+^ T cell density in variated distances from the vessels. Data in bar graphs are presented as mean ± SD, analyzed by One-way ANOVA. Each dot represents one mouse. *, *P* < 0.05; **, *P* < 0.01; ***, *P* < 0.001

We next examined the expression of intracellular adhesion molecule-1 (ICAM-1) on vascular endothelium, that interacts with LFA-1 on CD8^+^ T cells to facilitate the T cell extravasation [24]. In the tumors of colitis mice, surface expression of ICAM-1 on CD31^+^ vascular endothelial cells reduced significantly as comparison to that of normal mice. The decrease of ICAM-1 expression was associated with elevated IL-17A (Fig. 4A). Indeed, we observed the positive correlation between CD8^+^ T-cell infiltration and the expression levels of LFA-1 or ICAM-1 in certain types of human cancers in TCGA (Additional file 2: Fig. S8). The local generation of certain chemokines which regulate CD8^+^ T-cell extravasation and integrin expression was also examined. Transcription levels of *Cxcl9*, *Ccl3*, *Ccl4*, and *Ccl5*, which facilitate effector CD8^+^ T-cell extravasation into tissues [24], were significantly lower in the tumors of colitis mice than in that of normal mice (Fig. 4B). Multiplex staining validated the reduced ICAM-1 expression on tumor vascular endothelium of colitis mice (Fig. 4C). The density of CD31^+^ blood vessels was higher, but perivascular CD8^+^ T cells was less in the tumors of colitis mice than in that of normal mice (Fig. 4D). The reduction of CD8^+^ T cells mainly occurred in those within the 25 μm from the vasculature (Fig. 4E).

### IL-17A suppressed the stem-like exhausted CTL transmigration across vascular endothelium

**Fig. 5:**
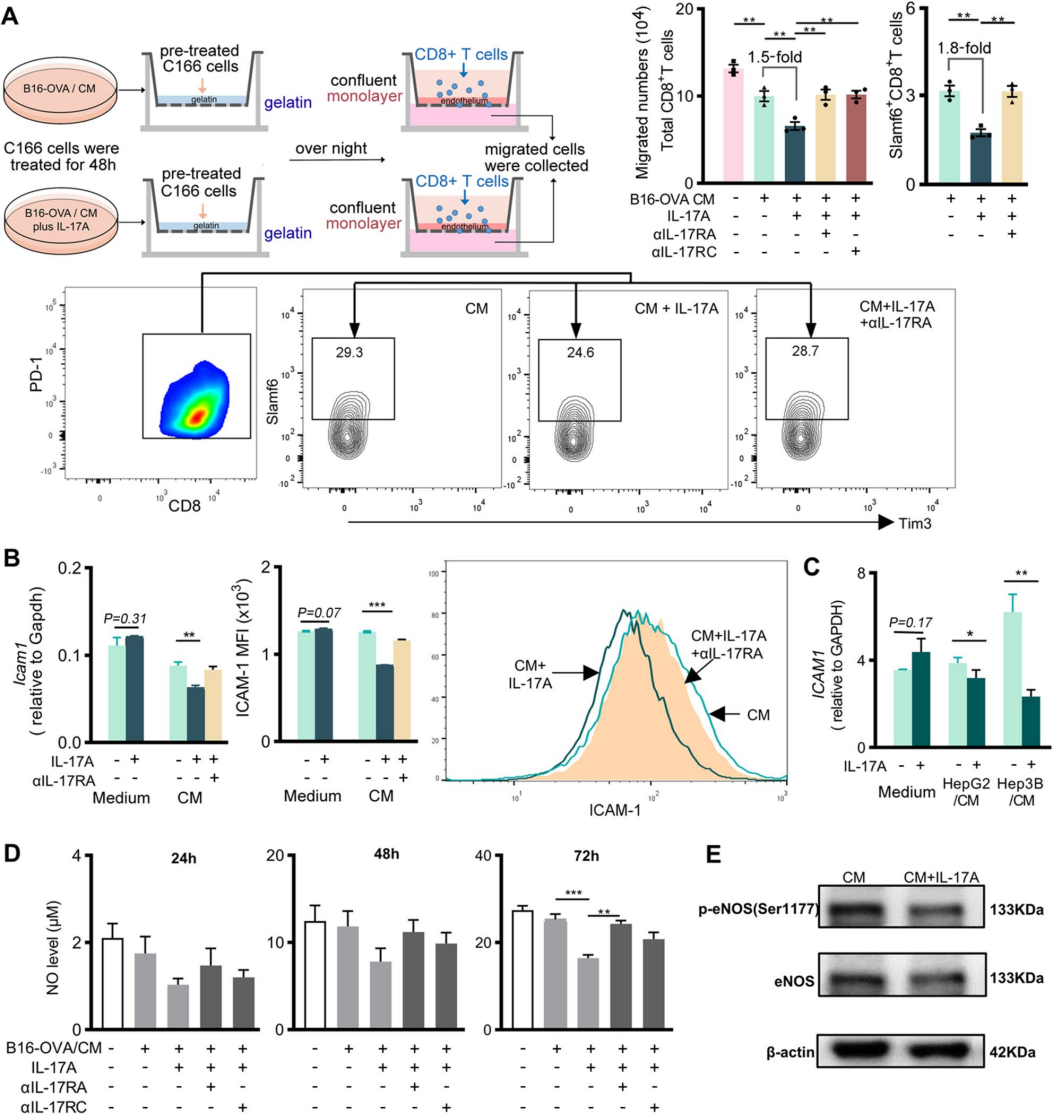
Effect of IL-17A on tumor-conditioned endothelial cells. **A** Experimental scheme for examining T cell transmigration across vascular endothelium. FCM profiles show one representative of three independent experiments. Bar graphs show the migrated numbers of total CD8^+^ T cells (left) and Slamf6^+^ stem-like subset (right) with indicated treatment. **B**, **C** ICAM-1 expression in differently treated C166 cells (**B**), HUVECs (**C**), for 48 h. **D** NO generation by C166 cells at indicated period. **E** Immunoblot images show the generation of eNOS, phosphorylated eNOS at Ser1177 in differently treated C166 cells for 48 h, β-actin as loading control. Data in bar graphs are presented as mean ± SD, compared with *t*-test (two groups) or One-way ANOVA (more than two groups). *, *P* < 0.05; **, *P* < 0.01; ***, *P* < 0.001

We next examined IL-17A effects on the interaction of CD8^+^ T cells with tumor vascular endothelium by using mouse C166 cells, that carry vascular endothelium characteristics [34] (depicted in Fig. 5A). CD8^+^ T cell adhesion to C166 cell monolayers treated with B16-OVA/CM displayed distinctly from the monolayers treated with regular medium after IL-17A was included (Additional file 2: Fig. S9A). Analysis on the public GSE51401 dataset, which examined the gene expression of CD31^+^ paired tumor endothelial cells (TECs) and non-tumor endothelial cells (NECs) from 16 HCC patients, indicated that the genes related to leukocyte trans-endothelial migration in TECs was suppressed as comparison with NECs (Additional file 2: Fig. S9B). When IL-17A was included into B16-OVA/CM, the CD8^+^ T cell transmigration was significantly inhibited. The inhibition on Slamf6^+^ T cell transmigration was more profound. Blocking IL-17A signaling through neutralizing IL-17RA (αIL-17RA) or neutralizing IL-17RC (αIL-17RC) restored the transmigration of total CD8^+^ T cells and the stem-like subset (Fig. 5A).

We then examined the effects of IL-17A on ICAM-1 expression in vascular endothelial cells. IL-17A addition into tumor cell-derived medium significantly reduced the ICAM-1 expression of both murine (C166 cells, Fig. 5B) and human (HUVECs, Fig. 5C) vascularly endothelial cells. Indeed, in the GSE51401 dataset, *ICAM1* transcription level was negatively correlated with *IL-17A* level of tumor tissues and with *IL17RC* in TECs but not in NECs (Additional file 2: Fig. S9C).

In addition to LFA-1 integrins, the extravasation of effector T cells from the blood into peripheral tissues depends on the normal function of vascular endothelium, which is crucially controlled by local generation of nitric oxide (NO) by endothelial cells [35]. As the reports of local NO generation in improving ICB-immunotherapy [36, 37], we examined the IL-17A on NO production from endothelial cells. Addition of IL-17A into the B16-OVA/CM significantly reduced the NO generation from C166 cells, and blocking the IL-17A signaling recovered the NO generation (Fig. 5D). Addition of IL-17A to the B16-OVA/CM reduced the production of stimulatory eNOS at serine 1177 phosphorylation (p-eNOS-Ser1177) to limit the NO production from C166 cells (Fig. 5E, the original images are shown in Additional file 3).

### Neutralizing IL-17A improved anti-PD-1 antitumor efficacy in a murine model

**Fig. 6:**
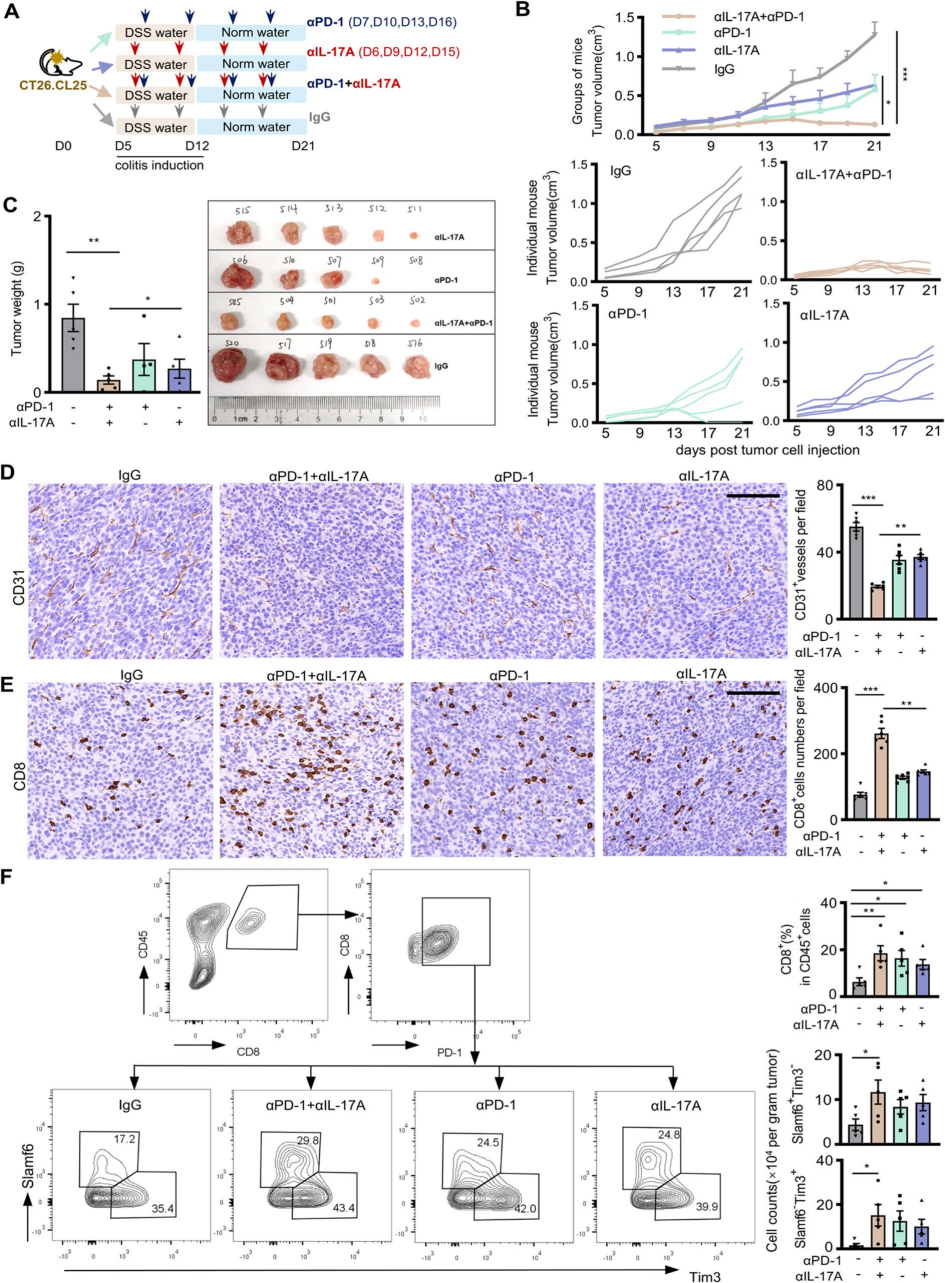
Blocking IL-17A on anti-PD-1 treatment effect in mice with colitis. **A** Experimental scheme, 5 mice were included in each group and repeated twice. All mice were sacrificed on D21. **B** Tumor growth as indicated. **C** Tumor weight on D21. Representative images for staining (brown) of CD31^+^ cells (**D**), CD8^+^ cells (**E**) in the tumors of indicated mice, scale bars, 100 μm. Specified cell density calculated from 2 fields/slide of each tumor are presented in bar graphs. **F** Infiltration of two CD8^+^ T cell subsets in the tumors of differently treated mice. FCM profiles show one representative of five independent repeats. Bar graphs show the average of the indicated group of mice. Data in bar graphs are presented as mean ± SD, compared with One-way ANOVA. Each dot represents one mouse. *, *P* < 0.05; **, *P* < 0.01; ***, *P* < 0.001

We inoculated CT26.CL25 cells into female BALB/c mice, induced colitis, and administrated neutralizing antibody against PD-1 (αPD-1), against IL-17A (αIL-17A), or against both (αPD-1 + αIL-17A) (Fig. 6A). Compared with the isotype-treated mice (IgG), tumor growth in the αPD-1-treated mice or the αIL-17A-treated mice was slower. Notably, tumor growth in the mice that received two antibodies (αPD-1 plus αIL-17A) was mostly inhibited (Fig. 6B and C). In formalin-fixed tumor tissues, the tumor angiogenesis indicated by CD31^+^ cell density reduced, and CD8^+^ T cell infiltration increased in the mice treated with αPD-1 alone or αIL-17A alone. Remarkably, the density of CD31^+^ vessel was profoundly lower, and CD8^+^ T cells were markedly increased in the tumors of mice that received αPD-1 plus αIL-17A (Fig. 6D and E). FCM analysis of the freshly-removed tumor tissues showed that the numbers of CD8^+^ T cells in the αPD-1 plus αIL-17A-treated mice enhanced most significantly. The proportion of Slamf6^+^Tim3^−^ stem-like subset displayed significantly higher in the tumors of mice that received αPD-1 plus αIL-17A than in the mice that received IgG (Fig. 6F).

### Post-therapy elevation of serum IL-17A in HCC patients resisted to anti-PD-1 plus anti-VEGF combined therapy

**Fig. 7:**
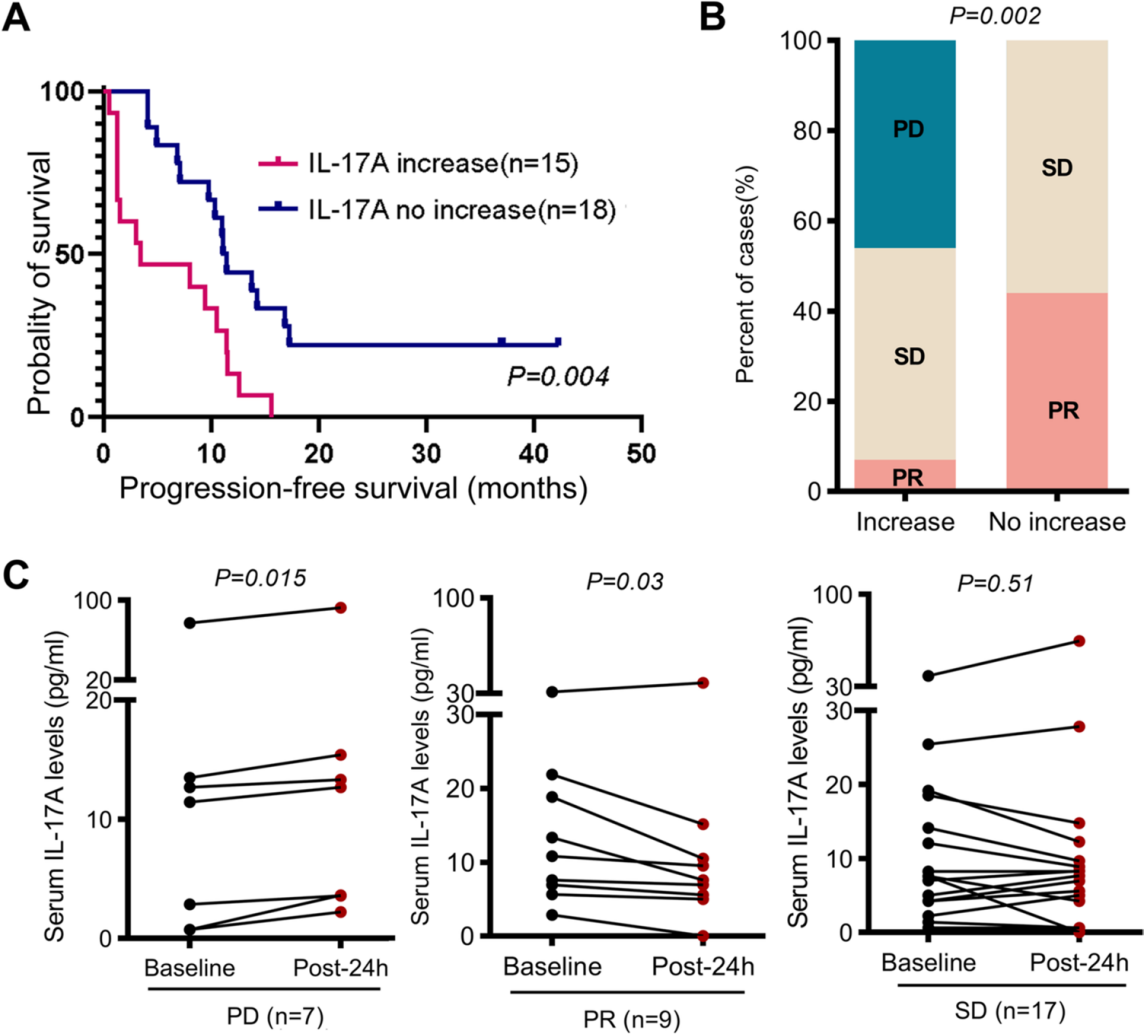
Alterations of post-therapy serum IL-17A on disease progression in advanced HCC patients that received anti-PD-1 plus anti-VEGF. **A** Serum IL-17A levels of 33 advanced HCC patients were quantified before and 24 h post the therapy. The mPFS in 15 patients with IL-17A increase (red line), and 18 patients with IL-17A no-increase (blue line) 24 h post-therapy, log-rank test. **B** Percentage of the patients with different disease progression, analyzed by Fisher’s exact test. **C** Serum IL-17A levels pre-therapy and 24 h post-therapy in each patient with different disease progression, paired *t*-test. PR, partial response; SD, stable disease; PD, progressive disease

We extended our observation to a cohort of advanced HCC patients that received the anti-PD-1 and anti-VEGF combined therapy [32]. In comparison to the pre-therapy serum IL-17A, 15 of 33 tested patients exhibited serum IL-17A increase, 18 patients no-increase (decrease or stable) 24 h post-therapy. The median progression-free survival (mPFS), time from the treatment initiation to disease progression, was only 3.4 months in the 15 patients, significantly shorter than the mPFS (11.3 months) of the other 18 patients (Fig. 7A). Only one of the 15 patients (6.7%) with IL-17A increase post-therapy exhibited disease partial response (PR), while 8/18 (44.4%) of the patients with IL-17A no-increase post-therapy demonstrated PR (Fig. 7B). All grades of gastrointestinal adverse events were reported by 6 patients, and only one of them experienced grade III diarrhea. All the patients with progressive disease (PD) tended to IL-17A elevation, while 8 of 9 patients (88.9%) with PR tended to IL-17A reduction 24 h post-therapy (Fig. 7C).

## Discussion

In this study, we uncovered a novel role of abnormally generated IL-17A on tumor infiltration of two exhausted CTL subsets (Additional file 2: Fig. S10). Stem-like CTLs were recognized to express higher IL-17RA and IL-17RC but lower LFA-1, as compared to the terminally exhausted CTLs. In tumor-bearing mice, colitis-related IL-17A significantly suppressed the extravasation and self-renewal of the stem-like subset, dampening CTL-conferred antitumor immunity. Neutralizing IL-17A improved tumor infiltration of “stem-like exhausted” CTLs and enhanced the anti-PD-1-mediated antitumor efficacy. The HCC patients with serum IL-17A elevation 24 h post-therapy displayed resistance to the combined therapy of anti-PD-1 plus anti-VEGF, displaying worse disease progression. ICB-immunotherapy relies on the intra-tumoral stem-like exhausted CTLs rather than the lone reversal of the exhausted program [21, 22]. Our current study indicated that IL-17A stimulation mainly suppressed the tumor infiltration of stem-like CTLs. Therefore, ICB-based immunotherapeutic efficacy could be upgraded by blocking IL-17A activities when treatment-related colitis occurred, even the adverse events were minor.

IL-17A carry out the function via signals through the IL-17RA and IL-17RC/ IL-17RD receptor subunits and the acts of IL-17A were well recognized in non-hematopoietic cells and innate immune cells [6, 33]. Recent investigation of IL-17A directly on T cells by using *Il17a*^−/−^ mice reported that IL-17A can promote terminal exhaustion of CD8^+^ T cells and tumor progression [38]. However, the exhausted CD8^+^ T cell population in the tumor tissues is heterogenous, and the stem-like exhausted CTL subset control tumor growth better than the terminally exhausted CTL subset does [21, 22]. Restricted expression of IL-17RC/IL-17RD in non-hematopoietic cells limits IL-17A signaling downstream [6, 33]. In this study, we detected higher surface expression of both IL-17RA and IL-17RC in the “stem-like exhausted” CTLs than that in the “terminally exhausted” CTLs, and validated by two public data GSE84105 and GSE123235 which quantified various gene transcription levels of in two exhausted CTL subsets [19, 22]. Our results indicated that the “stem-like exhausted” CTLs are more susceptible to IL-17A stimulation. Indeed, the tumor infiltration of the stem-like subset, not the terminal subset, reduced significantly in the colitis mice with abnormal IL-17A generation. Nevertheless, downstream molecular events after IL-17R heterodimer signaling in the specified CTLs need to be identified.

LFA-1 interaction with ICAM-1 on vascular endothelium is essential for effector T cell extravasation into peripheral tissues [24]. In the tumors of colitis mice, ICAM-I expression on the tumor blood vessels decreased, and perivascular CD8^+^ T cell density reduced, particularly those within 25 μm of the vasculature. In the cell culture system, IL-17A addition profoundly inhibited ICAM-I expression on endothelial cells and suppressed the transmigration of CTLs, particularly the stem-like subset, across the tumor vascular endothelium. On the stem-like CTLs, we detected lower levels of LFA-1 expression, and the surface LFA-1 expression was further reduced by colitis-induced IL-17A. While IL-17A showed minor effects on LFA-1 surface expression on the “terminally exhausted” CTLs. Tumor-associated high endothelial venules (TA-HEVs) were recognized as major sites of lymphocyte extravasation into tumors both at baseline and upon ICB treatment. MECA-79^+^ TA-HEVs display a unique phenotype, expressing certain adhesion molecules to facilitate the entry of some types of T cells via interacting with the counterpart molecules on the T cells [39]. Two exhausted CTL subsets express distinct cytokine receptors and adhesion molecules [19, 21, 22], implying that the two CTL subsets might have different entry gates into tumors. Our current results indicated that the cell-surface molecule LFA-1 is an important molecule regulated by IL-17A. The entry of stem-like CTLs into tumors was particularly restrained in response to the instigated cytokine IL-17A from the inflamed intestine. In addition to LFA-1, integrin VLA-4 (α4β1 integrin) expression of T cells, which binds to vascular cell adhesion molecule-1 (VCAM1) on endothelial cells, also controls effector T cell tumor infiltration [24]. It was reported that LFA-1 activation by a small-molecule activator of LFA-1 and VLA-4 improved ICB therapeutic efficacy by promoting T cell infiltration into the tumor microenvironment [40]. We observed no difference in α4 subunit transcription between two CTL subsets, but higher β1 subunit transcription in the stem-like subset. The impacts of IL-17A on VLA-4 in CD8^+^ T cell tumor infiltration require our further investigation.

To improve ICB-immunotherapy, targeting angiogenesis such as anti-VEGF, which was considered to inhibit the sprouting of new vessels and also “normalize” the tumor vasculature to improve immune cell infiltration, was combined with anti-PD-1/PD-L1 for the cancer treatment and obtained efficacious effects in HCC treatment [23, 25, 41]. Nevertheless, the effect of blocking VEGF alone displayed less efficacious than double-blocking VEGF and ANG2 for improving T cell tumor infiltration [31]. IL-17A is able to activate multiple IL-17R-expressing cell types to repress CD8^+^T cells mediated antitumor immunity [5, 7–14, 42]. IL-17A could activate myeloid-derived suppressor cells, recruit neutrophils, and induce the nuclear translocation of hypoxia-inducible factor-1α in cancer-associated fibroblasts to exclude CD8 + T cell infiltration [5, 7, 9, 42, 43]. IL-17A has also been demonstrated to promote tumor angiogenesis through promoting the generation of conventional VEGF or directly acting on the endothelial cells [7, 11–14, 23]. IL-17 was found directly acts on endothelial cells of the vasculature to decrease the NO production by inducing the phosphorylation of eNOS at the inhibitory site threonine 495 [44] and to participate in hypertension [45]. In our cell culture system, we detected that the production of stimulatory eNOS at serine 1177 from endothelial cells was reduced after IL-17A stimulation, resulting in decreased NO generation. The transmigration of stem-like CD8^+^ T cells was significantly inhibited. Results from our current study indicated that IL-17A stimulation exaggerates tumor vasculature dysfunction to restrain the extraversion of CTLs, particularly the stem-like CTLs, leading to uncontrol of tumor growth. Therefore, neutralizing IL-17A could recover the interaction of effector CD8^+^ T cells and tumor vascular endothelium via the LFA-1/ICAM-1, and also restore the NO production from endothelial cells, facilitating the infiltration of CD8^+^ T cells, particularly the stem-like subset, into the tumor bed. Local delivery of NO proves to improve the ICB-immunotherapy efficacy [35–37]. Clinical studies have reported that the use of angiotensin system inhibitors could improve immunotherapy [46].

Clinical studies pointed out the association between the onset of immune-related adverse events and better overall survival and overall response to ICB immunotherapy [47]. The hypothesis is being challenged. A study reported recently that colitis tissues of ICB-treated patients with immune-related enterocolitis (irEC) displayed significantly higher Th17 cell gene expression scores, compared to the paired normal intestinal tissues. Transcription of IL-6 cytokine increased by 11.7-folds, and IL-17A increased by 6.3-folds in the colitis tissues of ICB-treated patients with irEC. Their tumor-bearing mice models showed that IL-6 blockade improves ICB-induced antitumor efficacy. Clinical data indicated that targeting IL-6 can mitigate the immune-related adverse event without compromising the overall tumor response to ICB [15]. IL-6 is required for the retaining transcriptional and functional identity of Th17 cells [48]. In addition, IL-17A elevation also occurs in patients receiving chemotherapy of irradiation [16, 17]. Our current study indicated that abnormally generated IL-17A conferred more suppressive effects on the extravasation of ″stem-like'' CTLs to dampen the CD8^+^ T cell-mediated antitumor activity. Neutralizing IL-17A could restore the tumor infiltration of ''stem-like exhausted'' CTLs and enhance the anti-PD-1-mediated antitumor efficacy. We provided an option by neutralizing IL-17A to improve ICB-based therapeutic efficacy when abnormal IL-17A generation in the setting, such as colitis, occurred during cancer treatment.

## Conclusions

Stem-like exhausted CTLs express higher IL-17A rector heterodimers, susceptible to abnormally generated IL-17A. Stimulation of IL-17A diminishes the stem-like exhausted CTL interaction with tumor endothelium to repress the CTL extravasation and inhibited the CTL self-renewal in the tumor bed. An intervention approach for blocking abnormally generated IL-17A could upgrade the therapeutic efficacy conferred by immune checkpoint blockades.

### Supplementary Information

**Additional file 1: Supplementary Materials and Methods.** **Table S1.** List of antibodies and reagents. **Table S2.** List of primers used in quantitative real-time PCR. **Table S3.** Differentially expressed genes between CD31+ tumor endothelial cells and CD31+ non-tumor endothelial cells in GSE51401. **Table S4.** Demographics and baseline characteristics of the 33 HCC patients received a combined immunotherapy of anti-PD-1 plus anti-VEGF antibodies.

**Additional file 2: Fig. S1.** Antigen-specific CD8+T cell-mediated antitumor effects in murine tumor models with colitis. **Fig. S2.** IL-17A producing cells in inflamed intestinal tissue of murine tumor models with colitis. **Fig. S3.** Transcription levels of different IL-17A receptor subunits in two CTL subsets, Il17ra (A), Il17rc (B), and IlI7rd (C), based on public data. **Fig. S4.** Transcription levels of LFA-1 subunits and VLA-4 subunits in two CTL subsets based on public data. **Fig. S5.** The infiltration of two CTL subsets in the tumors of differently treated mice based on the surface markers of CXCR5 and Tim3. **Fig. S6.** IL-17A on proliferation of CTL subsets. **Fig. S7.** Effects of IL-17A on the cytokines release, cell apoptosis and tumor cytotoxicity of the antigen specific CD8+ T cells. **Fig. S8.** Correlation between the indicated markers of some human cancers in TCGA database, download from TCGA database on August 6, 2022. **Fig. S9.** Effect of IL-17A on tumor vascular endothelium. **Fig. S10.** Graphic abstract.

**Additional file 3. The original immunoblot images of Fig. 5E.**

## Data Availability

All the data supporting the conclusions of this study are included within the article and its additional files.

## Abbreviations

ANG2: Angiopoietin-2
ATCC: American Type Culture Collection
CTL: Cytotoxic T lymphocyte
CTLA-4: Cytotoxic T-lymphocyte-associated protein-4
DSS: Dextran sulfate sodium
eNOS: Endothelial NO synthase
FCM: Flow cytometry
HCC: Hepatocellular carcinoma
HUVEC: Human umbilical vein endothelial cell
ICAM-1: Intracellular adhesion molecule-1
ICB: Immune checkpoint blockade
IHC: Immunohistochemistry
IL-17A: Interleukin 17A
IL-17RA: Interleukin 17 receptor A subunit
IL-17RC: Interleukin 17 receptor C subunit
LFA-1: Leukocyte function-associated antigen-1
NEC: Non-tumor endothelial cell
NO: Nitric oxide
OVA: Ovalbumin
PD: Progressive disease
PD-1: Programmed death-1
PFS: Progression-free survival
PR: Partial response
SD: Stable disease
TA-HEV: Tumor-associated high endothelial venule
TEC: Tumor endothelial cell
TIL: Tumor infiltrated lymphocyte
VEGF: Vascular endothelial growth factor

## References

[1] Morad G, Helmink BA, Sharma P, Wargo JA (2021). Hallmarks of response, resistance, and toxicity to immune checkpoint blockade. Cell.

[2] Nielsen DL, Juhl CB, Chen IM, Kellermann L, Nielsen OH (2022). Immune checkpoint Inhibitor-Induced diarrhea and Colitis: Incidence and Management. A systematic review and Meta-analysis. Cancer Treat Rev.

[3] Ramos-Casals M, Brahmer JR, Callahan MK, Flores-Chavez A, Keegan N, Khamashta MA, Lambotte O, Mariette X, Prat A, Suarez-Almazor ME (2020). Immune-related adverse events of checkpoint inhibitors. Nat Rev Dis Primers.

[4] Leppkes M, Becker C, Ivanov II, Hirth S, Wirtz S, Neufert C, Pouly S, Murphy AJ, Valenzuela DM, Yancopoulos GD (2009). RORgamma-expressing Th17 cells induce murine chronic intestinal inflammation via redundant effects of IL-17A and IL-17F. Gastroenterology.

[5] Majumder S, McGeachy MJ (2021). IL-17 in the pathogenesis of disease: good intentions gone Awry. Annu Rev Immunol.

[6] Li X, Bechara R, Zhao J, McGeachy MJ, Gaffen SL (2019). IL-17 receptor-based signaling and implications for disease. Nat Immunol.

[7] Ma S, Cheng Q, Cai Y, Gong H, Wu Y, Yu X, Shi L, Wu D, Dong C, Liu H (2014). IL-17A produced by gammadelta T cells promotes tumor growth in hepatocellular carcinoma. Can Res.

[8] Coffelt SB, Kersten K, Doornebal CW, Weiden J, Vrijland K, Hau CS, Verstegen NJM, Ciampricotti M, Hawinkels L, Jonkers J (2015). IL-17-producing gammadelta T cells and neutrophils conspire to promote breast cancer metastasis. Nature.

[9] Li TJ, Jiang YM, Hu YF, Huang L, Yu J, Zhao LY, Deng HJ, Mou TY, Liu H, Yang Y (2017). Interleukin-17-Producing Neutrophils Link Inflammatory Stimuli to Disease Progression by Promoting Angiogenesis in Gastric Cancer. Clin Cancer Res.

[10] Zhang Y, Chandra V, Riquelme Sanchez E, Dutta P, Quesada PR, Rakoski A, Zoltan M, Arora N, Baydogan S, Horne W (2020). Interleukin-17-induced neutrophil extracellular traps mediate resistance to checkpoint blockade in pancreatic cancer. J Exp Med.

[11] Ribatti D (2019). Interleukins as modulators of angiogenesis and anti-angiogenesis in tumors. Cytokine.

[12] Chen X, Cai G, Liu C, Zhao J, Gu C, Wu L, Hamilton TA, Zhang CJ, Ko J, Zhu L (2019). IL-17R-EGFR axis links wound healing to tumorigenesis in Lrig1(+) stem cells. J Exp Med.

[13] Chen J, Ye X, Pitmon E, Lu M, Wan J, Jellison ER, Adler AJ, Vella AT, Wang K (2019). IL-17 inhibits CXCL9/10-mediated recruitment of CD8(+) cytotoxic T cells and regulatory T cells to colorectal tumors. J Immunother Cancer.

[14] Chung AS, Wu X, Zhuang G, Ngu H, Kasman I, Zhang J, Vernes JM, Jiang Z, Meng YG, Peale FV (2013). An interleukin-17-mediated paracrine network promotes tumor resistance to anti-angiogenic therapy. Nat Med.

[15] Hailemichael Y, Johnson DH, Abdel-Wahab N, Foo WC, Bentebibel SE, Daher M, Haymaker C, Wani K, Saberian C, Ogata D (2022). Interleukin-6 blockade abrogates immunotherapy toxicity and promotes tumor immunity. Cancer Cell.

[16] Zhong W, Li Q (2017). Rituximab or irradiation promotes IL-17 secretion and thereby induces resistance to rituximab or irradiation. Cell Mol Immunol.

[17] Bruchard M, Mignot G, Derangere V, Chalmin F, Chevriaux A, Vegran F, Boireau W, Simon B, Ryffel B, Connat JL (2013). Chemotherapy-triggered cathepsin B release in myeloid-derived suppressor cells activates the Nlrp3 inflammasome and promotes tumor growth. Nat Med.

[18] Li F, Li C, Cai X, Xie Z, Zhou L, Cheng B, Zhong R, Xiong S, Li J, Chen Z (2021). The association between CD8+ tumor-infiltrating lymphocytes and the clinical outcome of cancer immunotherapy: a systematic review and meta-analysis. EClinicalMedicine.

[19] Im SJ, Hashimoto M, Gerner MY, Lee J, Kissick HT, Burger MC, Shan Q, Hale JS, Lee J, Nasti TH (2016). Defining CD8+ T cells that provide the proliferative burst after PD-1 therapy. Nature.

[20] Utzschneider DT, Charmoy M, Chennupati V, Pousse L, Ferreira DP, Calderon-Copete S, Danilo M, Alfei F, Hofmann M, Wieland D (2016). T Cell Factor 1-expressing memory-like CD8(+) T cells sustain the immune response to chronic viral infections. Immunity.

[21] Siddiqui I, Schaeuble K, Chennupati V, Fuertes Marraco SA, Calderon-Copete S, Pais Ferreira D, Carmona SJ, Scarpellino L, Gfeller D, Pradervand S (2019). Intratumoral Tcf1(+)PD-1(+)CD8(+) T Cells with Stem-like Properties Promote Tumor Control in Response to Vaccination and Checkpoint Blockade Immunotherapy. Immunity.

[22] Miller BC, Sen DR, Al Abosy R, Bi K, Virkud YV, LaFleur MW, Yates KB, Lako A, Felt K, Naik GS (2019). Subsets of exhausted CD8(+) T cells differentially mediate tumor control and respond to checkpoint blockade. Nat Immunol.

[23] Fukumura D, Kloepper J, Amoozgar Z, Duda DG, Jain RK (2018). Enhancing cancer immunotherapy using antiangiogenics: opportunities and challenges. Nat Rev Clin Oncol.

[24] Vestweber D (2015). How leukocytes cross the vascular endothelium. Nat Rev Immunol.

[25] Finn RS, Qin S, Ikeda M, Galle PR, Ducreux M, Kim TY, Kudo M, Breder V, Merle P, Kaseb AO (2020). Atezolizumab plus Bevacizumab in Unresectable Hepatocellular Carcinoma. N Engl J Med.

[26] Chen K, Wu Z, Zhao H, Wang Y, Ge Y, Wang D, Li Z, An C, Liu Y, Wang F (2020). XCL1/Glypican-3 fusion gene immunization generates potent antitumor cellular immunity and enhances anti-PD-1 efficacy. Cancer Immunol Res.

[27] Perse M, Cerar A (2012). Dextran sodium sulphate colitis mouse model: traps and tricks. J Biomed Biotechnol.

[28] Nambiar DK, Aguilera T, Cao H, Kwok S, Kong C, Bloomstein J, Wang Z, Rangan VS, Jiang D, von Eyben R (2019). Galectin-1-driven T cell exclusion in the tumor endothelium promotes immunotherapy resistance. J Clin Investig.

[29] Xing X, Yang J, Yang X, Wei Y, Zhu L, Gao D, Li M (2013). IL-17A induces endothelial inflammation in systemic sclerosis via the ERK signaling pathway. PLoS One.

[30] Sun Z, Nyberg R, Wu Y, Bernard B, Redmond WL (2021). Developing an enhanced 7-color multiplex IHC protocol to dissect immune infiltration in human cancers. PLoS One.

[31] Schmittnaegel M, Rigamonti N, Kadioglu E, Cassara A, Wyser Rmili C, Kiialainen A, Kienast Y, Mueller HJ, Ooi CH (2017). Laoui D *et al*: Dual angiopoietin-2 and VEGFA inhibition elicits antitumor immunity that is enhanced by PD-1 checkpoint blockade. Sci Transl Med.

[32] Zhang W, Gong C, Peng X, Bi X, Sun Y, Zhou J, Wu F, Zeng H, Wang Y, Zhou H (2022). Serum Concentration of CD137 and Tumor Infiltration by M1 Macrophages Predict the Response to Sintilimab plus Bevacizumab Biosimilar in Advanced Hepatocellular Carcinoma Patients. Clin Cancer Res.

[33] Su Y, Huang J, Zhao X, Lu H, Wang W, Yang XO, Shi Y, Wang X, Lai Y, Dong C (2019). Interleukin-17 receptor D constitutes an alternative receptor for interleukin-17A important in psoriasis-like skin inflammation. Sci Immunol.

[34] Wang SJ, Greer P, Auerbach R (1996). Isolation and propagation of yolk-sac-derived endothelial cells from a hypervascular transgenic mouse expressing a gain-of-function fps/fes proto-oncogene. In Vitro Cell Dev Biol Anim.

[35] Lundberg JO, Weitzberg E (2022). Nitric oxide signaling in health and disease. Cell.

[36] Sung YC, Jin PR, Chu LA, Hsu FF, Wang MR, Chang CC, Chiou SJ, Qiu JT, Gao DY, Lin CC (2019). Delivery of nitric oxide with a nanocarrier promotes tumour vessel normalization and potentiates anti-cancer therapies. Nat Nanotechnol.

[37] Kim J, Francis DM, Sestito LF, Archer PA, Manspeaker MP, O'Melia MJ, Thomas SN (2022). Thermosensitive hydrogel releasing nitric oxide donor and anti-CTLA-4 micelles for anti-tumor immunotherapy. Nat Commun.

[38] Kim BS, Kuen DS, Koh CH, Kim HD, Chang SH, Kim S, Jeon YK, Park YJ, Choi G, Kim J (2021). Type 17 immunity promotes the exhaustion of CD8(+) T cells in cancer. J Immunother Cancer.

[39] Asrir A, Tardiveau C, Coudert J, Laffont R, Blanchard L, Bellard E, Veerman K, Bettini S, Lafouresse F, Vina E (2022). Tumor-associated high endothelial venules mediate lymphocyte entry into tumors and predict response to PD-1 plus CTLA-4 combination immunotherapy. Cancer Cell.

[40] Hickman A, Koetsier J, Kurtanich T, Nielsen MC, Winn G, Wang Y, Bentebibel SE, Shi L, Punt S, Williams L (2022). LFA-1 activation enriches tumor-specific T cells in a cold tumor model and synergizes with CTLA-4 blockade. J Clin Invest.

[41] Feng ZY, Xu FG, Liu M, Xu HJ, Wu FB, Chen HB, Xia HP (2021). The immune microenvironment and progression of immunotherapy and combination therapeutic strategies for hepatocellular carcinoma. Hepatoma Res.

[42] Chen X, Zhao J, Herjan T, Hong L, Liao Y, Liu C, Vasu K, Wang H, Thompson A, Fox PL (2022). IL-17-induced HIF1alpha drives resistance to anti-PD-L1 via fibroblast-mediated immune exclusion. J Exp Med.

[43] Patil RS, Shah SU, Shrikhande SV, Goel M, Dikshit RP, Chiplunkar SV (2016). IL17 producing gammadeltaT cells induce angiogenesis and are associated with poor survival in gallbladder cancer patients. Int J Cancer.

[44] Nguyen H, Chiasson VL, Chatterjee P, Kopriva SE, Young KJ, Mitchell BM (2013). Interleukin-17 causes Rho-kinase-mediated endothelial dysfunction and hypertension. Cardiovasc Res.

[45] Davis GK, Fehrenbach DJ, Madhur MS (2021). Interleukin 17A: Key Player in the Pathogenesis of Hypertension and a Potential Therapeutic Target. Curr Hypertens Rep.

[46] Pinter M, Jain RK (2017). Targeting the renin-angiotensin system to improve cancer treatment: Implications for immunotherapy. Sci Transl Med.

[47] Das S, Johnson DB (2019). Immune-related adverse events and anti-tumor efficacy of immune checkpoint inhibitors. J Immunother Cancer.

[48] Harbour SN, DiToro DF, Witte SJ, Zindl CL, Gao M, Schoeb TR, Jones GW, Jones SA, Hatton RD, Weaver CT (2020). T(H)17 cells require ongoing classic IL-6 receptor signaling to retain transcriptional and functional identity. Science immunology.

