# Supplementary material for: Abnormal generation of IL-17A represses tumor infiltration of stem-like exhausted CD8+ T cells to demote the antitumor immunity
Source: BMC Med. 2023 Aug 21;21:315. doi: 10.1186/s12916-023-03026-y (PMC10441727; doi:10.1186/s12916-023-03026-y)
Supplement: Supplementary file 1 — Additional file 1: Supplementary Materials and Methods. Table S1. List of antibodies and reagents. Table S2. List of primers used in quantitative real-time PCR. Table S3. Differentially expressed genes between CD31+ tumor endothelial cells and CD31+ non-tumor endothelial cells in GSE51401. Table S4. Demographics and baseline characteristics of the 33 HCC patients received a combined immunotherapy of anti-PD-1 plus anti-VEGF antibodies. [file 12916_2023_3026_MOESM1_ESM.docx]

**Additional file 1 for**

**Abnormal generation of IL-17A represses tumor infiltration of stem-like exhausted CD8+ T cells to demote the antitumor immunity**

Ruochan Zhang, Kun Chen*, Caifeng Gong, Zhiyuan Wu, Chungui Xu, Xing-Ning Li, Fei Zhao, Dongmei Wang, Jianqiang Cai, Aiping Zhou*, and Chunfeng Qu*

**Supplementary materials and methods.**

**Quantification of cytokines in intestine tissues and serum**

Interstitial fluid levels of inflammatory cytokines and chemokines were quantified by using the Quansys Mouse assay kit and software (Quansys Biosciences, Utah, USA) according to the manufacture's instruction. Serum IL-17A levels were quantified using a commercialized Mouse IL-17A ELISA Kit (Andygene, Beijing, China）.

**Immunohistochemistry (IHC)**

The IHC was conducted in paraffin-embedded tissue sections using standard laboratory protocols. Slides were scanned with Pannoramic MIDI slide scanner (3DHISTECH, Budapest, Hungary) at 20 × magnification, analyzed with ImageJ software (Bethesda, Maryland, USA). The information of used antibodies is listed in **Table S1.**

**Flow cytometry (FCM)**

Tumor tissues were treated in the solution containing 1 mg/mL collagenase D and 0.02 mg/mL DNase I (Sigma, MO, USA). Intestine tissues were treated in the solution containing 500 U/mL collagenase Ⅲ,1 M Hepes and 0.02 mg/mL DNase I. All samples were firstly stained with Live/Dead dye (Biolegend, CA, USA) for 20 min at room temperature, following addition of the antibodies to specified cell markers. The information of used antibodies is listed in **Table S1.**

**Table S1**. List of antibodies and reagents

| Antibody/Reagent | Application | Manufacturer | Location | Catalog # | Dilution |
| --- | --- | --- | --- | --- | --- |
| **Antibody** | | | | | |
| IL-17A | IHC | Proteintech | Shanghai, China | 26163-1-AP | 1:100 |
| CD8 | mIHC | Abcam | Cambridge, MA, USA | ab217344 | 1:2000 |
| Ki67 | mIHC | Abcam | Cambridge, MA, USA | ab279653 | 1:2000 |
| CD31 | mIHC | Abcam | Cambridge, MA, USA | ab281583 | 1:4000 |
| ICAM-1 | mIHC | Abcam | Cambridge, MA, USA | ab17907 | 1:4000 |
| eNOS | Immunoblot | Abcam | Cambridge, MA, USA | ab199956 | 1:1000 |
| p-eNOS Ser1177 | Immunoblot | Abcam | Cambridge, MA, USA | ab215717 | 1:1000 |
| β-actin | Immunoblot | Proteintech | Shanghai, China | 66009-1-Ig | 1:2000 |
| IL-17A | ICFC | eBioscience | San Diego, CA, USA | 17-7177-81 |  |
| Ki67 | ICFC | Biolegend | San Diego, CA, USA | 652425 |  |
| CD45.1 | FCM | eBioscience | San Diego, CA, USA | 11-0453 |  |
| CD45.2 | FCM | eBioscience | San Diego, CA, USA | 12-0454 |  |
| CD3 | FCM | eBioscience | San Diego, CA, USA | 12-0031 |  |
| CD8 | FCM | eBioscience | San Diego, CA, USA | 17-0081 |  |
| CD4 | FCM | eBioscience | San Diego, CA, USA | 12-0041 |  |
| γδ TCR | FCM | eBioscience | San Diego, CA, USA | 11-5811 |  |
| CD45 | FCM | Biolegend | San Diego, CA, USA | 157214 |  |
| CD31 | FCM | Biolegend | San Diego, CA, USA | 160206 |  |
| PD-1 | FCM | Biolegend | San Diego, CA, USA | 135216 |  |
| Slamf6 | FCM | Biolegend | San Diego, CA, USA | 134608 |  |
| CXCR5 | FCM | Biolegend | San Diego, CA, USA | 145526 |  |
| TIM-3 | FCM | Biolegend | San Diego, CA, USA | 134012 |  |
| ICAM-1 | FCM | Biolegend | San Diego, CA, USA | 116122 |  |
| IL-17A | Animal | BioXcell | West Lebanon, NH, USA | BE0173 |  |
| PD-1 | Animal | BioXcell | West Lebanon, NH, USA | BE0273 |  |
| IgG isotype | Animal | BioXcell | West Lebanon, NH, USA | BE0083 |  |
| IL-17RA | NEU | R&D Systems | Minneapolis, MN, USA | MAB4481 |  |
| IL-17RC | NEU | R&D Systems | Minneapolis, MN, USA | AF2270 |  |
| **Reagent** | | | | | |
| DSS | Animal | MP Biomedicals | Irvine, CA, USA | 02160110 |  |
| CFSE | ICFC | eBioscience | San Diego, CA, USA | C34554 |  |
| IL-17A ELISA Kit | ELISA | Andy gene | Beijing, China | AD3394Mo |  |
| NO assay kit | ELISA | Beyotime | Shanghai, China | S0021S |  |
| Phosphatase inhibitors | Immunoblot | Proteintech | Zhejiang, China | PR20015 |  |
| Protease inhibitors | Immunoblot | Roche | Basel, Switzerland | 04693116001 | |
| OVA_257-264_ peptide | Cell culture | Chinese Peptide | Zhejiang, China | MISC-012 |  |
| gp100_25-33_ peptide | Cell culture | Chinese Peptide | Zhejiang, China | Custom synthesis | |
| Recombinant mouse IL-17A | Cell culture | Peprotech | Minneapolis, MN, USA | 210-17 |  |
| *****Recombinant human IL-17A | Cell culture | Peprotech | Minneapolis, MN, USA | 200-17 |  |

*The reagent was used in human system, all the others list here were used in mouse system.

IHC, Immunohistochemistry; mIHC, Multiplex immunohistochemistry; ICFC, intracellular flow cytometry; FCM, flow cytometry; NEU, neutralization;

**Examination of gene expression using** **quantitative real-time PCR**

Total mRNA from tumor tissue samples or differently treated cells were extracted using TRIzol (Invitrogen, CA, USA) following the manufacture’s instruction. The cDNA was synthesized using a high-capacity cDNA reverse-transcription kit, and the specified gene transcription was determined using a SYBR Premix Ex Taq II kit (Takara Bio, Tokyo, Japan). The primer sequences are listed in **Table S2.**

**Table S2**. List of primers used in quantitative real-time PCR

| **Gene** | **Orientation** | **Sequence, 5’ to 3’** |
| --- | --- | --- |
| Human *ICAM1* | Forward | TTGGGCATAGAGACCCCGTT |
|  | Reverse | GCACATTGCTCAGTTCATACACC |
| Human *GAPDH* | Forward | GCACCGTCAAGGCTGAGAAC |
|  | Reverse | TGGTGAAGACGCCAGTGGA |
| Mouse *Icam1* | Forward | GTGATGCTCAGGTATCCATCCA |
|  | Reverse | CACAGTTCTCAAAGCACAGCG |
| Mouse *Cxcl9* | Forward | GGAGTTCGAGGAACCCTAGTG |
|  | Reverse | GGGATTTGTAGTGGATCGTGC |
| Mouse *Ccl3* | Forward | TTCTCTGTACCATGACACTCTGC |
|  | Reverse | CGTGGAATCTTCCGGCTGTAG |
| Mouse *Ccl4* | Forward | TTCCTGCTGTTTCTCTTACACCT |
|  | Reverse | CTGTCTGCCTCTTTTGGTCAG |
| Mouse *Ccl5* | Forward | GCTGCTTTGCCTACCTCTCC |
|  | Reverse | TCGAGTGACAAACACGACTGC |
| Mouse *Gapdh* | Forward | AGGTCGGTGTGAACGGATTTG |
|  | Reverse | TGTAGACCATGTAGTTGAGGTCA |

**Public data analysis**

On Aug 6, 2022 we download the data sets of colorectal adenocarcinoma (COAD), skin cutaneous melanoma (SKCM), liver hepatocellular carcinoma (LIHC) and lung squamous cell carcinoma (LUSC) from the TCGA. The correlations between specified molecules were performed with the TIMER 2.0 database (https://cistrome.shinyapps.io/timer/). We obtained the RNA-Seq data of ‘stem-like exhausted’ and ‘terminally exhausted’ CD8+ T cells from Gene Expression Omnibus (GEO) at GSE84105 and GSE123235, and the data of tumor vascularly endothelial cells (TEC) and non-tumor vascularly endothelial cells (NEC) from GSE51401. The differentially expressed genes between CD31^+^ TECs and CD31^+^ NECs list in **Table S3.**

| **Table S3**. Differentially expressed genes between CD31^+^ tumor endothelial cells and CD31^+^ non-tumor endothelial cells in GSE51401 | | | | |
| --- | --- | --- | --- | --- |
| gene | logFC | logCPM | PValue | FDR |
| *ABCG5* | 0.8552 | 5.6149 | 4.94E-05 | 0.0602 |
| *ADAMTS2* | -0.7033 | 5.7255 | 3.44E-04 | 0.1897 |
| *AK093362* | 1.0407 | 5.1038 | 1.20E-04 | 0.1227 |
| *ANKRD20A1* | -0.8720 | 5.0790 | 1.28E-03 | 0.4419 |
| *APELA* | 0.7560 | 4.7263 | 2.72E-02 | 1.0000 |
| *ARNTL2-AS1* | 0.7343 | 4.8935 | 1.60E-02 | 1.0000 |
| *BMPER* | -1.3192 | 5.4999 | 7.59E-09 | 0.0001 |
| *BMX* | -0.7676 | 5.7085 | 1.36E-04 | 0.1227 |
| *C17orf74* | 0.7889 | 4.8767 | 1.05E-02 | 1.0000 |
| *C19orf80* | 0.7102 | 5.7604 | 3.31E-04 | 0.1897 |
| *C7* | -0.9859 | 5.6977 | 1.38E-06 | 0.0036 |
| *CAP2* | 0.7234 | 5.6981 | 3.81E-04 | 0.2037 |
| *CLEC1B* | -1.0728 | 5.9308 | 9.01E-09 | 0.0001 |
| *CLEC4M* | -1.0862 | 5.8675 | 1.57E-08 | 0.0001 |
| *CLGN* | 0.7157 | 5.6554 | 4.99E-04 | 0.2303 |
| *COX8C* | 1.1876 | 4.7369 | 4.95E-04 | 0.2303 |
| *CRISP3* | -0.7273 | 5.5876 | 5.72E-04 | 0.2492 |
| *CT47A1* | 0.9175 | 5.2731 | 2.73E-04 | 0.1826 |
| *CXCR1* | -0.7352 | 5.8688 | 8.11E-05 | 0.0908 |
| *DTHD1* | -0.8115 | 5.2615 | 1.09E-03 | 0.4009 |
| *DUSP5P1* | 0.7715 | 5.0920 | 4.93E-03 | 0.8848 |
| *ERVMER61-1* | 0.7201 | 4.7750 | 2.49E-02 | 1.0000 |
| *FCN2* | -0.9138 | 5.7351 | 5.73E-06 | 0.0123 |
| *FCN3* | -0.9630 | 6.1684 | 6.00E-09 | 0.0001 |
| *FGF20* | -0.7998 | 5.1904 | 2.42E-03 | 0.6318 |
| *FLJ13744* | 0.7431 | 5.2063 | 4.65E-03 | 0.8679 |
| *FSTL5* | 0.7574 | 5.1744 | 4.50E-03 | 0.8598 |
| *GAGE1* | 0.7151 | 5.6453 | 5.99E-04 | 0.2543 |
| *GAGE12B* | 0.7189 | 5.5182 | 1.37E-03 | 0.4676 |
| *GOLT1A* | 0.7758 | 5.7123 | 1.36E-04 | 0.1227 |
| *GPM6A* | -0.8486 | 5.6846 | 3.50E-05 | 0.0485 |
| *GPR1-AS* | 0.7758 | 5.2837 | 1.67E-03 | 0.5028 |
| *GPR158* | 0.7063 | 5.3034 | 4.30E-03 | 0.8514 |
| *HNF4A-AS1* | 0.7066 | 5.0867 | 1.11E-02 | 1.0000 |
| *HYDIN* | 0.9365 | 4.6628 | 7.62E-03 | 1.0000 |
| *IGSF11* | 0.7247 | 4.7440 | 2.86E-02 | 1.0000 |
| *IL13RA2* | -0.8886 | 5.7224 | 9.98E-06 | 0.0176 |
| *INMT* | -1.0267 | 5.6732 | 6.98E-07 | 0.0021 |
| *ISX* | 0.7346 | 5.2279 | 3.91E-03 | 0.8327 |
| *ITLN1* | -0.7699 | 6.0564 | 1.05E-05 | 0.0176 |
| *KANK4* | 0.7937 | 5.2178 | 1.56E-03 | 0.4952 |
| *LINC00645* | 0.8224 | 4.8937 | 6.11E-03 | 0.9703 |
| *LINC00648* | 0.7735 | 4.9309 | 1.11E-02 | 1.0000 |
| *LINC01021* | 0.9732 | 4.9070 | 1.71E-03 | 0.5099 |
| *LINC01419* | 0.8722 | 5.4669 | 1.81E-04 | 0.1419 |
| *LINC01426* | 0.7297 | 5.0822 | 8.15E-03 | 1.0000 |
| *LOC100130964* | 1.0352 | 4.7221 | 3.37E-03 | 0.7824 |
| *LOC100288966* | 0.7397 | 4.9555 | 1.12E-02 | 1.0000 |
| *LOC100505498* | 0.7576 | 5.3667 | 1.48E-03 | 0.4822 |
| *LOC100505718* | 0.8370 | 4.8080 | 1.06E-02 | 1.0000 |
| *LOC101927363* | 0.9431 | 4.7750 | 4.33E-03 | 0.8514 |
| *LOC101930028* | 0.9991 | 4.8074 | 1.93E-03 | 0.5540 |
| *LTF* | -0.7797 | 5.7629 | 8.75E-05 | 0.0935 |
| *LYVE1* | -0.7079 | 6.1463 | 2.20E-05 | 0.0324 |
| *MAGEA1* | 0.8197 | 5.4800 | 2.87E-04 | 0.1826 |
| *MAGEA2* | 0.9626 | 5.3827 | 5.12E-05 | 0.0602 |
| *MAGEA3* | 0.7515 | 5.6055 | 6.13E-04 | 0.2543 |
| *MAGEA6* | 0.7364 | 5.6367 | 5.60E-04 | 0.2492 |
| *MAP7D2* | 0.8979 | 5.0853 | 1.45E-03 | 0.4788 |
| *MGP* | -0.8129 | 5.8424 | 1.60E-05 | 0.0251 |
| *MME* | -0.7394 | 5.7885 | 1.39E-04 | 0.1227 |
| *MMP12* | 0.7334 | 6.2847 | 4.11E-06 | 0.0097 |
| *MMRN1* | -0.7043 | 5.8096 | 2.70E-04 | 0.1826 |
| *NDST3* | -0.9037 | 5.7170 | 6.84E-06 | 0.0134 |
| *NPW* | 0.9112 | 4.5643 | 1.35E-02 | 1.0000 |
| *NPY1R* | -1.0797 | 5.6301 | 2.94E-07 | 0.0010 |
| *OIT3* | -0.9422 | 5.9721 | 1.96E-07 | 0.0008 |
| *RGS7BP* | -0.7592 | 5.4388 | 9.44E-04 | 0.3641 |
| *RP11-171N4.1* | 0.8169 | 4.5714 | 2.83E-02 | 1.0000 |
| *RP11-486A14.1* | 0.9027 | 5.1286 | 8.88E-04 | 0.3540 |
| *RP3-525N10.2* | -0.8756 | 5.3039 | 4.06E-04 | 0.2122 |
| *RSPO2* | -0.8227 | 4.8794 | 8.05E-03 | 1.0000 |
| *SIAH3* | -0.7021 | 4.8365 | 3.08E-02 | 1.0000 |
| *SMPX* | 0.7062 | 5.1235 | 7.33E-03 | 1.0000 |
| *SP5* | 0.7326 | 5.1279 | 5.73E-03 | 0.9446 |
| *ST8SIA6-AS1* | 0.8304 | 5.4532 | 3.14E-04 | 0.1849 |
| *TMEM100* | -0.7331 | 5.5885 | 5.72E-04 | 0.2492 |
| *TNMD* | -1.4234 | 5.1823 | 8.46E-08 | 0.0004 |
| *VSTM1* | -0.7886 | 5.5406 | 2.39E-04 | 0.1713 |

**Table S4**. Demographics and baseline characteristics of the 33 HCC patients received a combined immunotherapy of anti-PD-1 plus anti-VEGF antibodies

| **Characters** | **Numbers (%)** |
| --- | --- |
| Sex |  |
| Male | 27 (82%) |
| Female | 6 (18%) |
| Age, median (25%-75%) | 56 (38-75) |
| < 60 | 14 (42%) |
| ≥60 | 19 (58%) |
| Barcelona Clinic Liver Cancer stage |  |
| C | 33 |
| Treatment lines |  |
| 1 | 33 |
| Dose group (IBI305) |  |
| 7.5mg/kg | 20 (61%) |
| 15mg/kg | 13 (39%) |
| Viral status |  |
| Hepatitis B virus^a^ | 31 (94%) |
| Hepatitis C virus | 0 |
| Uninfected | 2 (6%) |
| Baseline Alpha-fetoprotein concentration |  |
| ≥ 400ng/ml | 13 (39%) |
| ＜400ng/ml | 20 (61%) |
| Macrovascular invasion |  |
| Yes | 7 (21%) |
| No | 26 (79%) |
| Extrahepatic disease |  |
| Yes | 21 (64%) |
| No | 12 (36%) |
| Liver cirrhosis |  |
| Yes | 23 (70%) |
| No | 10 (30%) |
| Overall response |  |
| Partial response | 9 (27%) |
| Stable disease | 17 (52%) |
| Progressive disease | 7 (21%) |
| Median progression-free survival (month, 25%-75%) | 10.7 (8.7, 12.7) |

^a^HBV infection: hepatitis B surface antigen positive and/or detectable HBV DNA in serum;

HCV infection: hepatitis C antibody positive and detectable HCV RNA in serum.

**Note.**

During the treatment, three patients reported diarrhea (grade 1, 2, 3 of each). One patient who experienced grade III diarrhea displayed persistent high-level of serum IL-17A post-therapy and progressive disease.
