# Supplementary material for: Abnormal generation of IL-17A represses tumor infiltration of stem-like exhausted CD8+ T cells to demote the antitumor immunity
Source: BMC Med. 2023 Aug 21;21:315. doi: 10.1186/s12916-023-03026-y (PMC10441727; doi:10.1186/s12916-023-03026-y)
Supplement: Supplementary file 2 — Additional file 2: Fig. S1. Antigen-specific CD8+T cell-mediated antitumor effects in murine tumor models with colitis. Fig. S2. IL-17A producing cells in inflamed intestinal tissue of murine tumor models with colitis. Fig. S3. Transcription levels of different IL-17A receptor subunits in two CTL subsets, Il17ra (A), Il17rc (B), and IlI7rd (C), based on public data. Fig. S4. Transcription levels of LFA-1 subunits and VLA-4 subunits in two CTL subsets based on public data. Fig. S5. The infiltration of two CTL subsets in the tumors of differently treated mice based on the surface markers of CXCR5 and Tim3. Fig. S6. IL-17A on proliferation of CTL subsets. Fig. S7. Effects of IL-17A on the cytokines release, cell apoptosis and tumor cytotoxicity of the antigen specific CD8+ T cells. Fig. S8. Correlation between the indicated markers of some human cancers in TCGA database, download from TCGA database on August 6, 2022. Fig. S9. Effect of IL-17A on tumor vascular endothelium. Fig. S10. Graphic abstract. [file 12916_2023_3026_MOESM2_ESM.docx]

**Additional file 2 for**

**Abnormal generation of IL-17A represses tumor infiltration of stem-like exhausted CD8+ T cells to demote the antitumor immunity**

Ruochan Zhang, Kun Chen*, Caifeng Gong, Zhiyuan Wu, Chungui Xu, Xing-Ning Li, Fei Zhao, Dongmei Wang, Jianqiang Cai, Aiping Zhou*, and Chunfeng Qu*


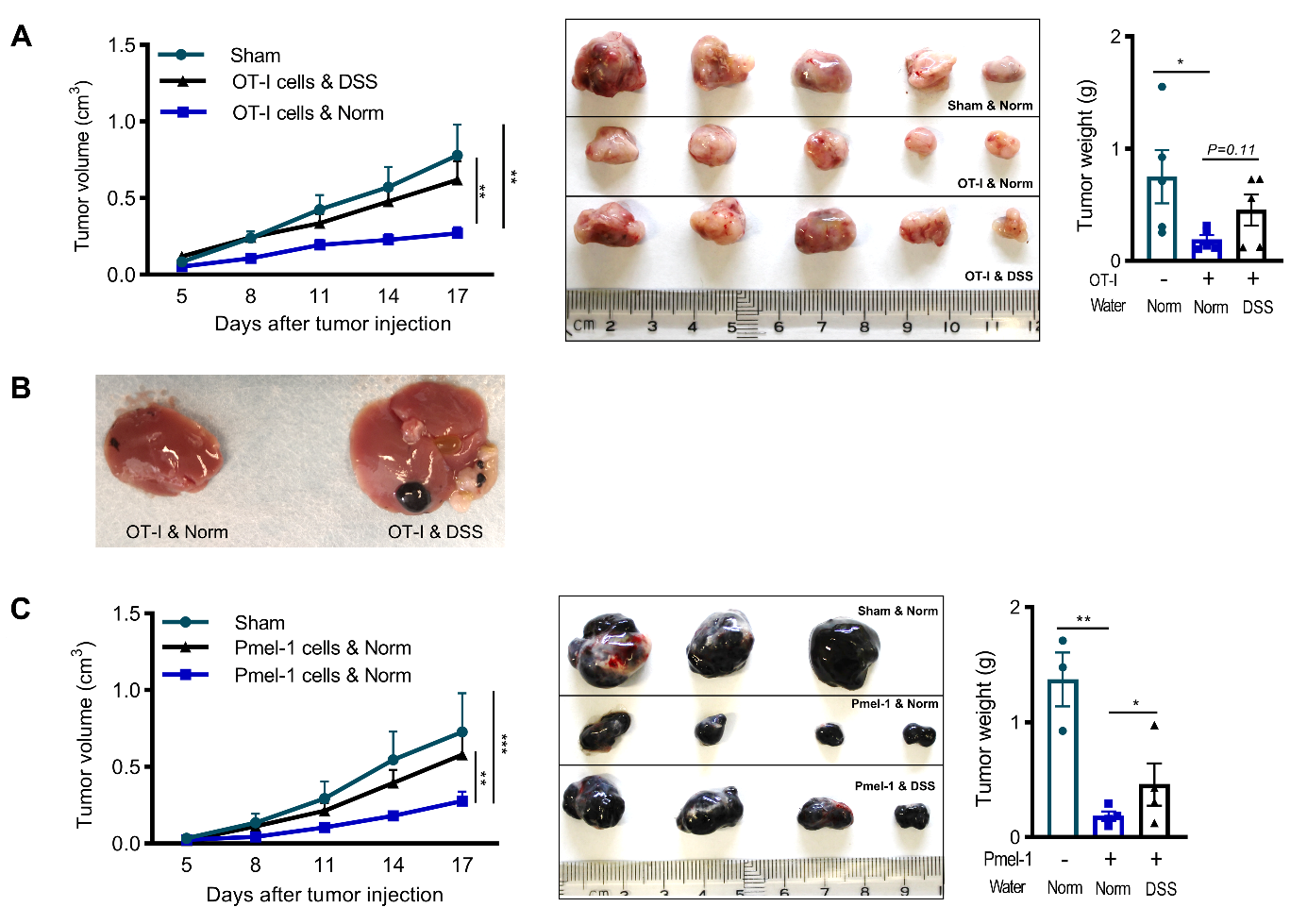


**Fig. S1. Antigen-specific CD8+T cell-mediated antitumor effects in murine tumor models with colitis**

**(A)** In each female C57BL/6 mouse, 3 × 10^6^ Hepa1-6-OVA cells were injected subcutaneously into the right flank. On D5, some mice (n=10) received activated OVA_257-264_-specific CD8+ T (OT-I) cells via tail vein injection, 1 × 10^6^ cells per mouse. Following cell transfer, 5 mice were given normal water consistently (OT-I & Norm), and 5 mice were given 2% DSS water for 1 week and then normal water (OT-I & DSS). As control, 5 tumor-bearing mice were injected intravenously with PBS and fed normal water (Sham). All mice were sacrificed on D18 when tumors in sham-treated group reached ≥2.0 cm in length. Tumor growth curve and tumor weight show as indicated. **(B)** In each female C57BL/6 mice, 5 × 10^5^ B16-OVA cells were inoculated into the left lateral lobe of mouse liver and received activated OT-I cells (2 × 10^6^ cells per mice) via tail vein injection on day 5. Following cell transfer, 3 mice were given normal water consistently, and 3 mice were given 2% DSS water for 1 week and then normal water. The mice were sacrificed by day 18. **(C)** In female C57BL/6 mice, 5 × 10^5^ B16-F10 cells were injected subcutaneously into the right flank. On D5, some mice (n=8) received activated GP100-specific CD8+ T (Pmel-1) cells via tail vein injection, 1 × 10^6^ cells per mouse. Following cell transfer, 4 mice were given normal water consistently (Pmel-1 & Norm), and 4 mice were given 2% DSS water for 1 week and then normal water (Pmel-1 & DSS). As control, 4 tumor-bearing mice were injected intravenously with PBS and fed normal water (Sham). All mice were sacrificed on D18 when tumors in sham-treated group reached ≥2.0 cm in length. Tumor growth curve and tumor weight show as indicated. In bar graphs, the data are presented as mean ± SD, each dot represent one mouse. Differences were compared by one-way ANOVA with Tukey’s multiple comparisons. *, *P <* 0.05; **, *P <* 0.01.

**
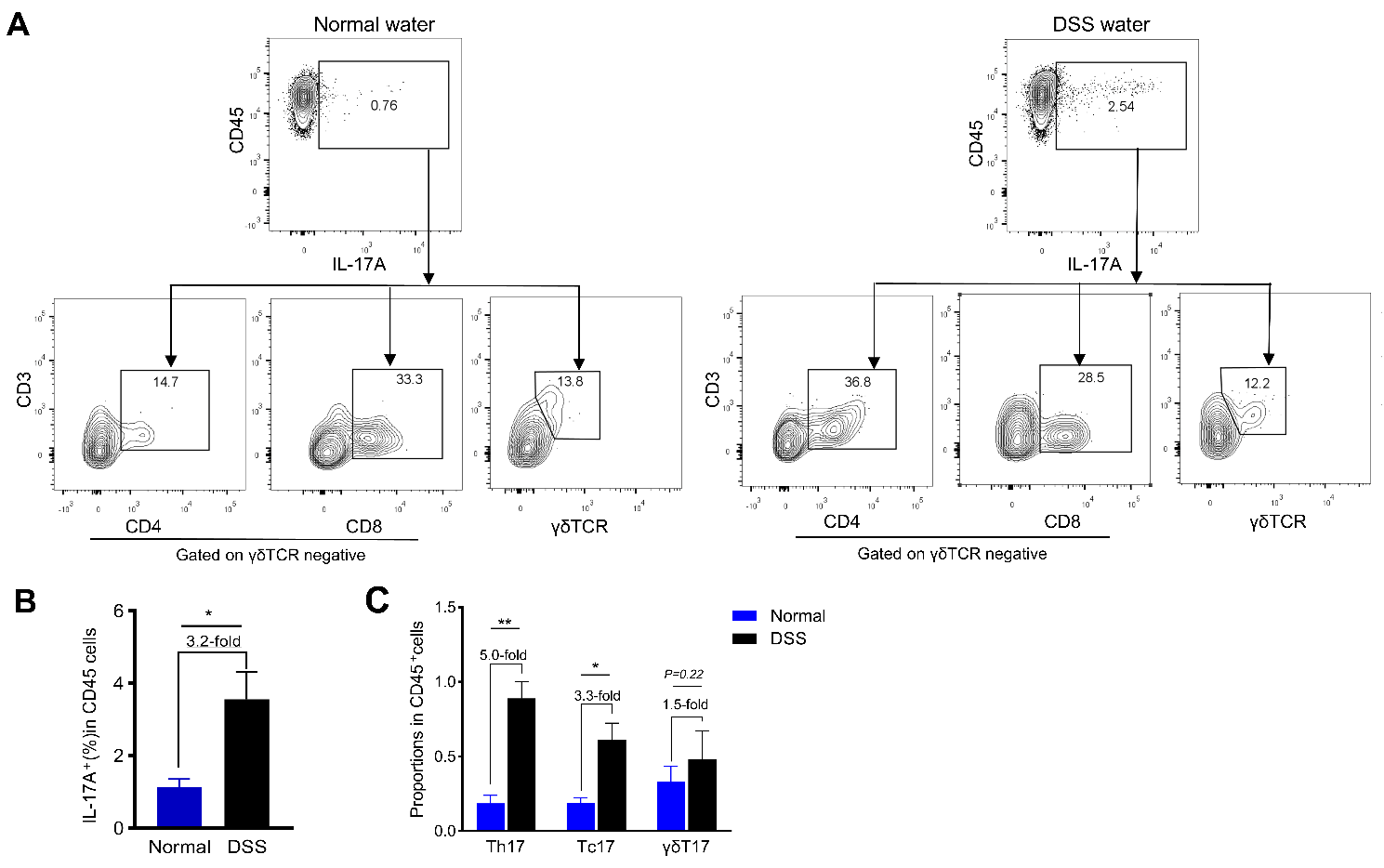
**

**Fig. S2. IL-17A producing cells in inflamed intestinal tissue of murine tumor models with colitis**

**(A)** FCM profiles show one representative of three independent experiments for determination of IL-17A-producing cells in inflamed intestinal tissue. **(B)** Percentage of IL-17A^+^ cells in total CD45^+^ immune cells of differently treated mice intestinal tissue. **(C)** The proportions of CD4^+^, CD8^+^, and γδTCR^+^ cells in the IL-17A-producing cells determined on D18 in indicated group of mice. Data are presented as mean ± SD, compared by two-tailed Student’s *t-test*. *, *P <* 0.05; **, *P <* 0.01.

**
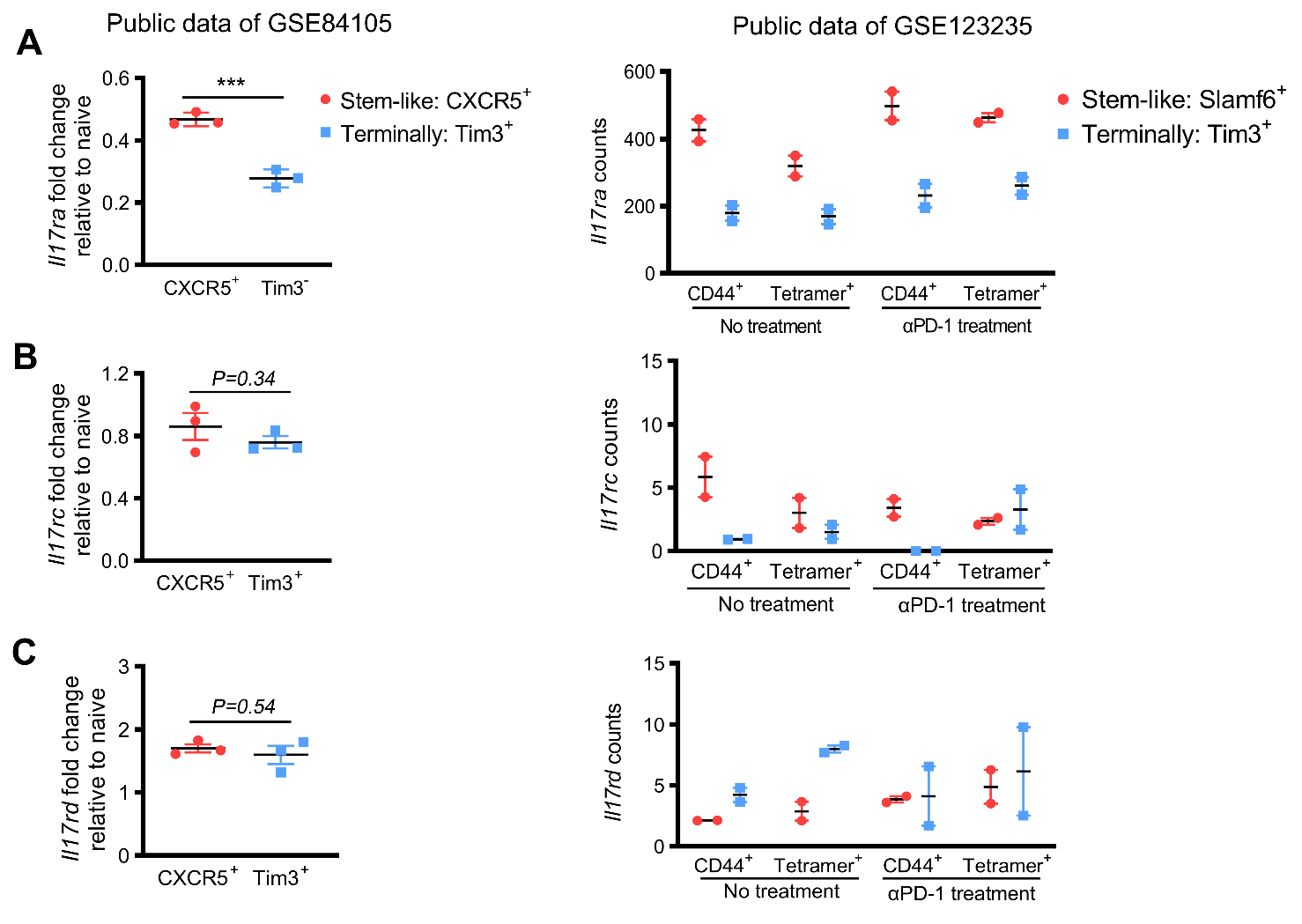
**

**Fig. S3.** **Transcription levels of different IL-17A receptor subunits in two CTL subsets,** ***Il17ra* (A)*, Il17rc* (B), and *IlI7rd* (C), based on public data**

In the GSE84105, data are shown as fold changes relative to naive CD8^+^ T cells and compared by Student’s *t*-test. ***, *P <* 0.001. In the GSE123235, the data indicate specified gene counts, each dot represents one of the independent replicates. No statistics was able to conduct.

**
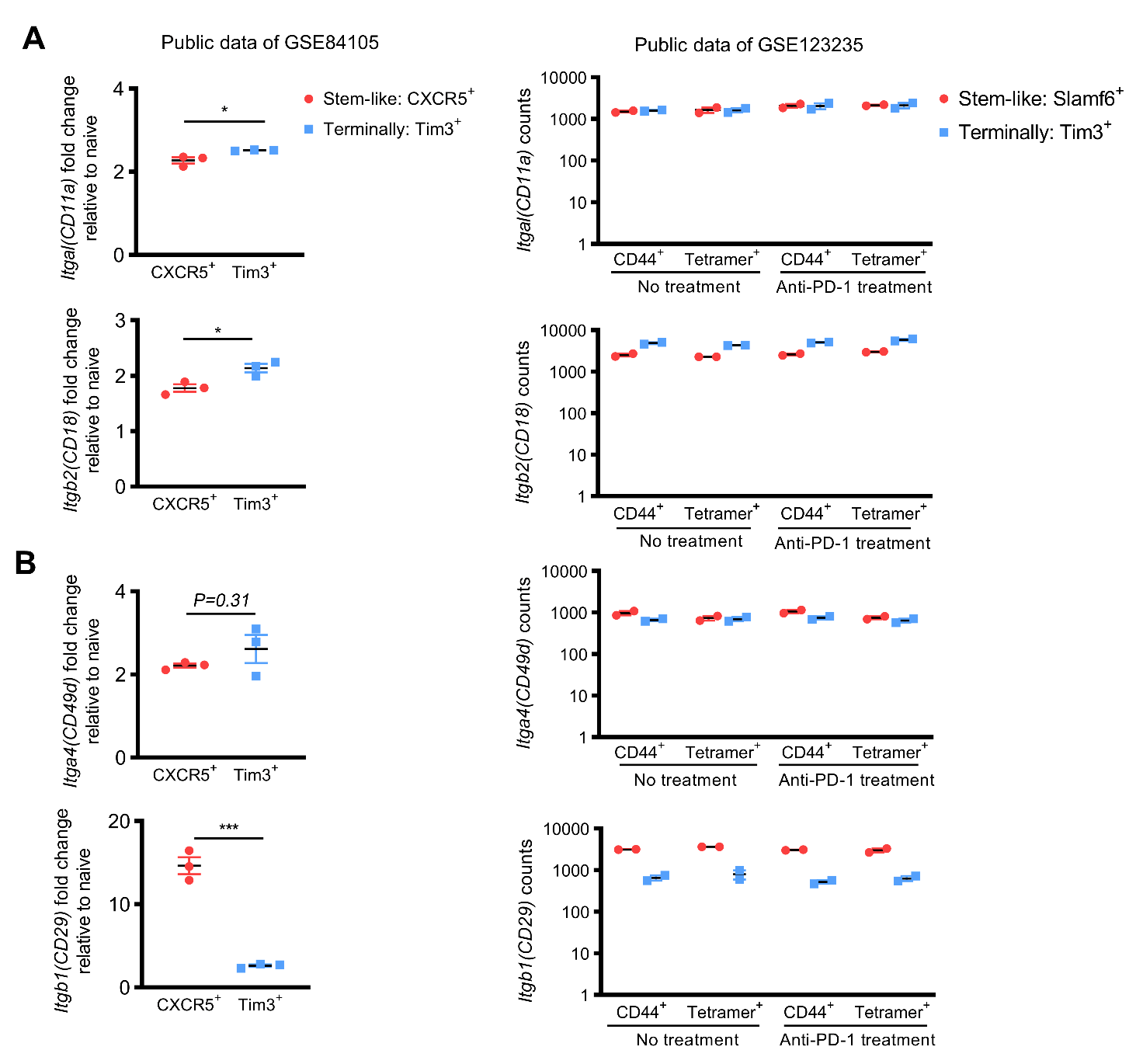
**

**Fig. S4.** **Transcription levels of LFA-1 subunits and VLA-4 subunits in two CTL subsets based on public data**

**(A)** Transcription levels of two LFA-1 subunits, *Itgal (CD11a)*, *Itgb2 (CD18)*; **(B)** transcription levels two VLA-4 subunits *Itga4(CD49d)*, *Itgb1(CD29)*. In the GSE84105, data are shown as fold changes relative to naive CD8+ T cells and compared by Student’s t-test. *, P < 0.05; ***, P < 0.001. In the GSE123235, the data indicate specified gene counts, each dot represents one of the independent replicates. No statistics was able to conduct.

**
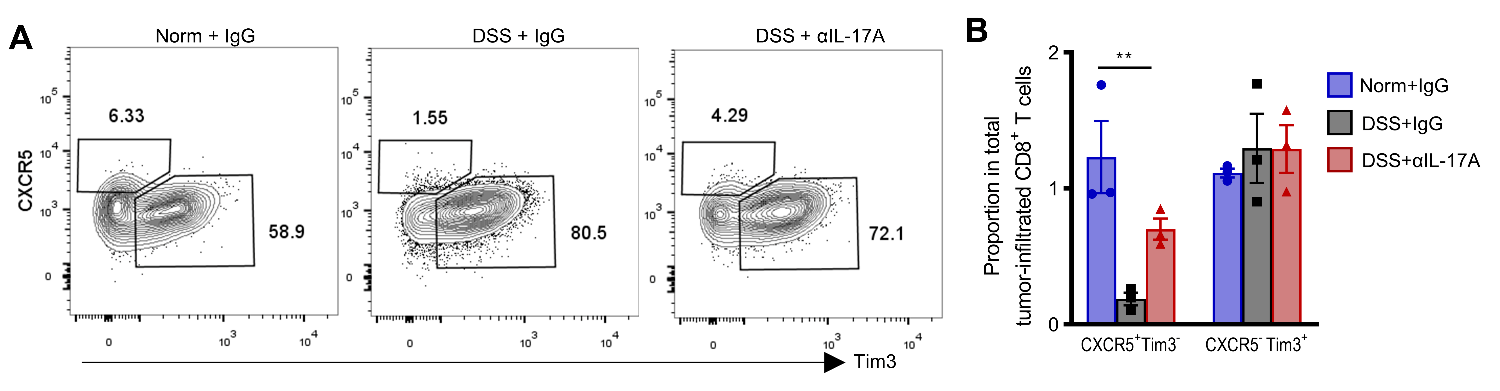
**

**Fig. S5. The infiltration of two CTL subsets in the tumors of differently treated mice based on the surface markers of CXCR5 and Tim3**

**(A)** FCM profiles show one representative of three independent experiments based on the surface expression of CXCR5^+^Tim3^-^ (stem-like subset) and CXCR5^-^Tim3^+^ (terminal subset). **(B)** The average of three independent experiments. Data indicate the ratio relative to normal mice (mean ± SD), analyzed by one-way ANOVA with Turkey’s multiple comparisons. **, *P <* 0.01.

**
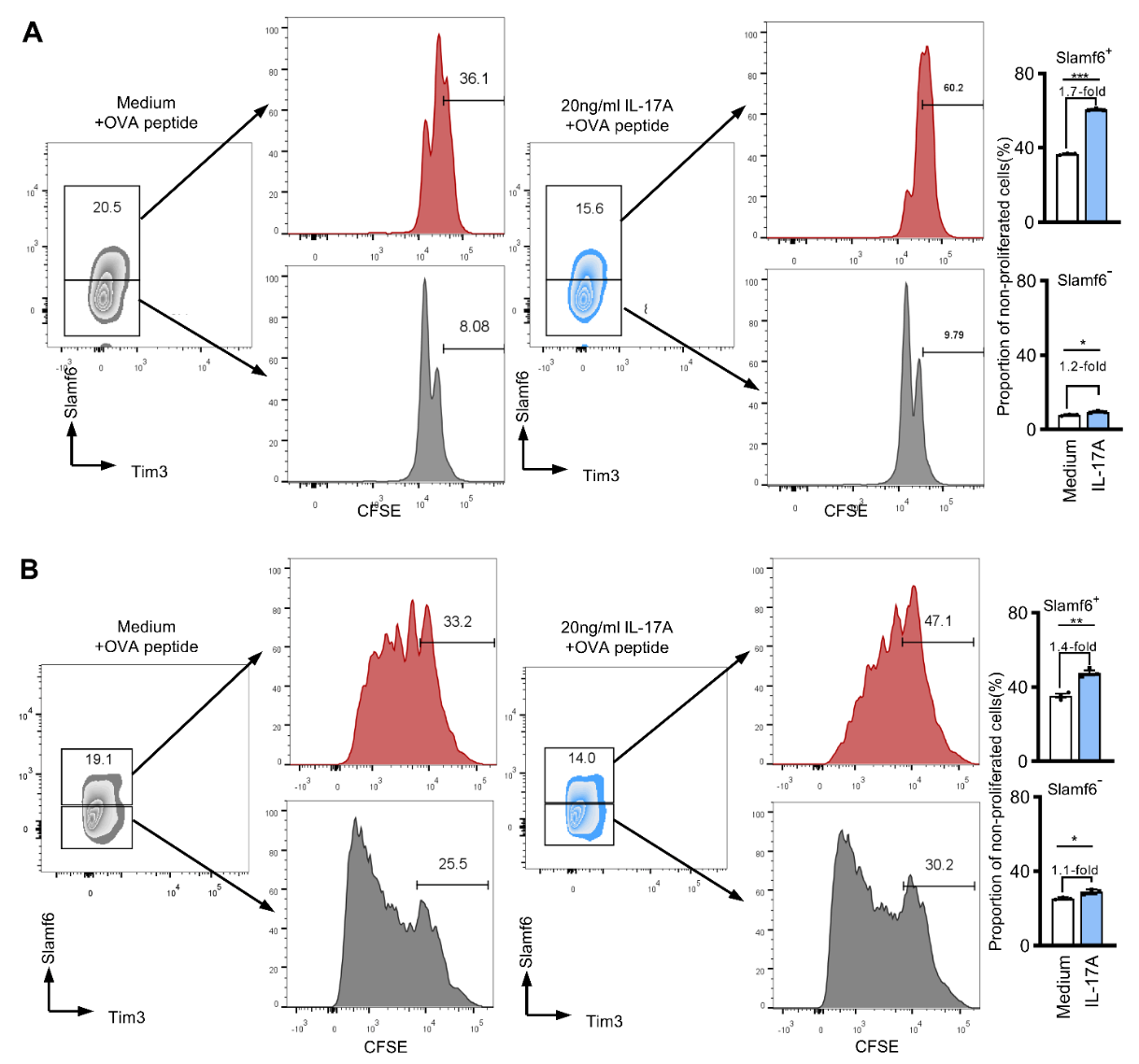
**

**Fig. S6**. **IL-17A on proliferation of CTL subsets**

OT-I cells were labeled with CFSE and stimulated with OVA_257-264_ peptide alone (medium) or supplement of 20 ng/ml of rmIL-17A. The proliferation was determined after being stimulated for 48h **(A)**, and 72h **(B)**. Each experiment was triplicated. Bar graphs show the mean ± SD and compared by Student’s t-test. *, P < 0.05; **, P < 0.01; ***, P < 0.001.


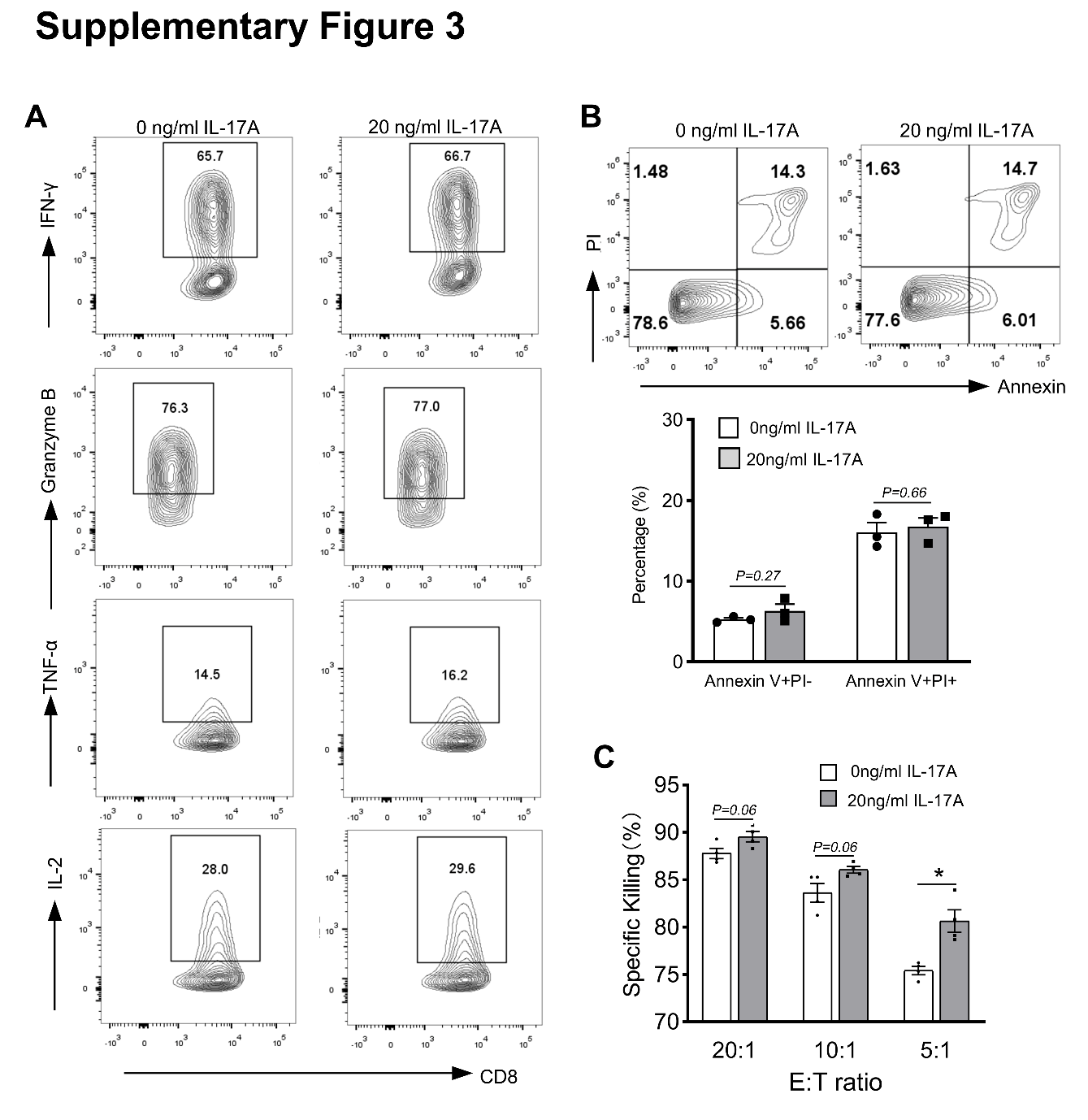


**Fig. S7. Effects of IL-17A on the cytokine release, cell apoptosis and tumor cytotoxicity of the antigen specific CD8^+^ T cells**

OT-I CD8^+^ T cells were cultured in the presence of OVA_257-264_ peptide, treated with or without recombinant mouse IL-17A (20 ng/ml) for 72 h. **(A)** FCM profiles show one representative of CD8^+^ T cells that producing IFN-γ, or Granzyme B, or TNF-α, or IL-2. **(B)** FCM profiles show one representative of CD8^+^ T cell apoptosis. **(C)** B16-OVA cells were labelled with 2 μM CSFE for 15 min and used as the target cells. At the indicated ratios, target cells were co-culture with OVA_257-264_-activated OT-I cells for 4 hours. The cells were collected and stained with PI for 5 min followed by FCM analysis. Specific killing percentage is calculated as = (% of dead target cells - % of spontaneously dead target cells) ×100 / (100 - % of spontaneously dead target cells). Data are presented as mean ± SD and compared with Student’s *t-*test. *, *P* < 0.05.

**
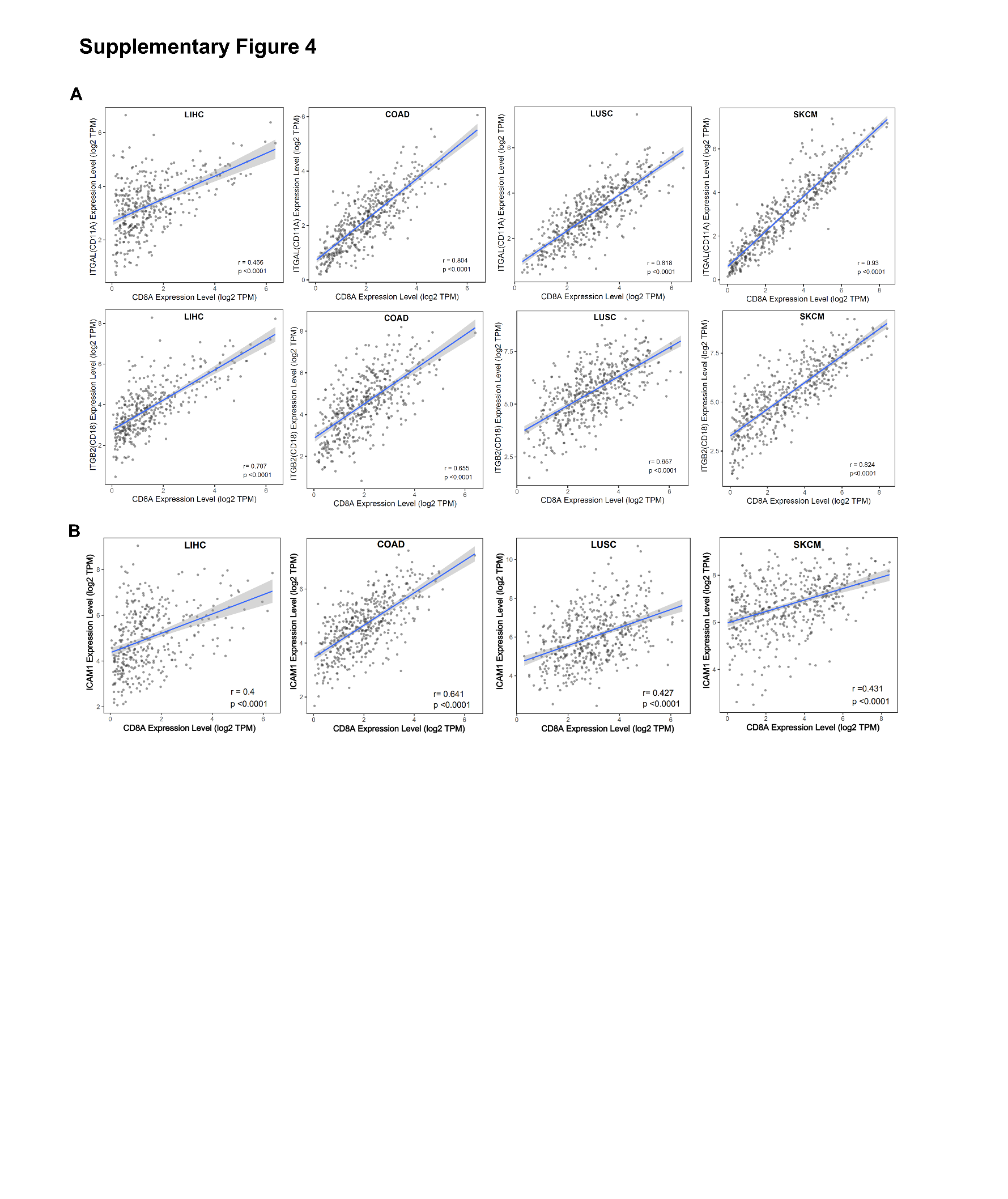
**

**Fig. S8. Correlation between the indicated markers of some human cancers in TCGA database, download from TCGA database on August 6, 2022**

The correlations were estimated using TIMER 2.0 database (<https://cistrome.shinyapps.io/timer/>).

**(A)** The correlation between the expression levels of CD8A and LFA-1 subunits ITGAL (CD11A), ITGB2 (CD18); **(B)** between the CD8A and ICAM1; in human liver hepatocellular carcinoma (LIHC), colorectal adenocarcinoma (COAD), lung squamous cell carcinoma (LUSC) and malignant melanoma (SKCM). TPM, transcripts Per Kilobase of exon model per Million mapped reads.

**
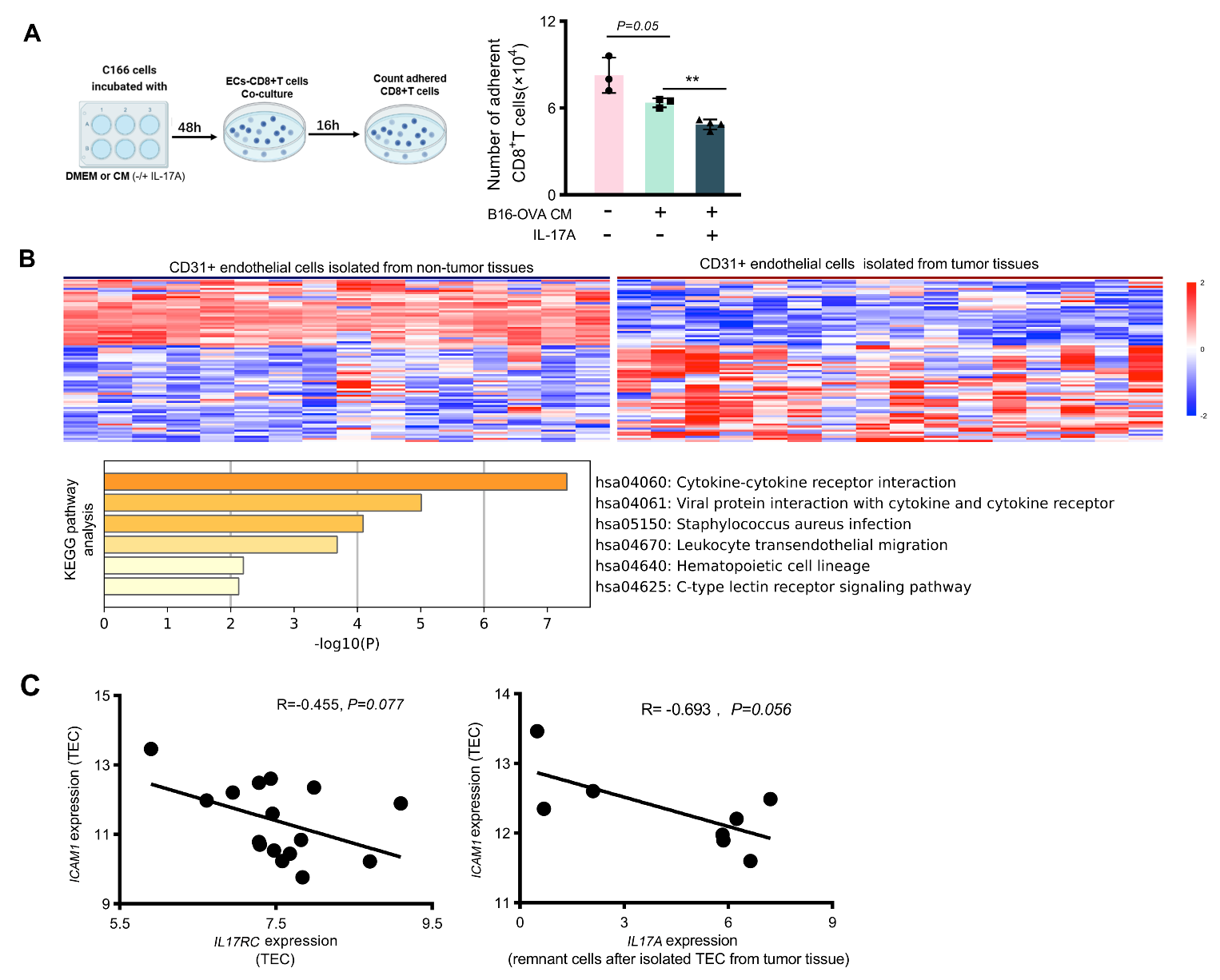
**

**Fig. S9. Effect of IL-17A on tumor vascular endothelium**

**(A)** Activated OT-I CD8^+^ T cells (2× 10^5^) were co-cultured with pretreated C166 cells (1× 10^5^) for 16 h. Quantification of CD8^+^ T cells adhered to C166 cells. Data are presented as mean ± SEM and analyzed by one-way ANOVA with Turkey’s multiple comparisons. **(B)** Gene expression data were obtained from GEO dataset GSE51401. Analysis of differentially expressed gene (DEG) between the CD31^+^ tumor endothelial cells (TEC) and CD31^+^ non-tumor endothelial cells (NEC) was performed using EdgeR_3.40.2. Significant DEGs were defined as **|** log2(Fold Change) **|** > 0.7 and P value < 0.05. Heatmaps were created using pheatmap_1.0.12 from row_scaled. Kyoto Encyclopedia of Genes and Genomes (KEGG) pathway enrichment analysis for CD31^+^ TEC downregulated DEGs by metascape online website (https://metascape.org). P Value Cut-off is 0.01 for the significance enrichment. The genes with significant differences are provided in **Supplementary Table S3**. **(C)**. Expression correlation between *ICAM1* and *IL17RC* in TEC (paired samples, n=16); and between *ICAM1* in TEC and *IL17A* in tumor cells (paired samples, n=8) in the public GEO dataset GSE51401. Pearson’s correlation test was used for statistics significance. **, *P <* 0.01.


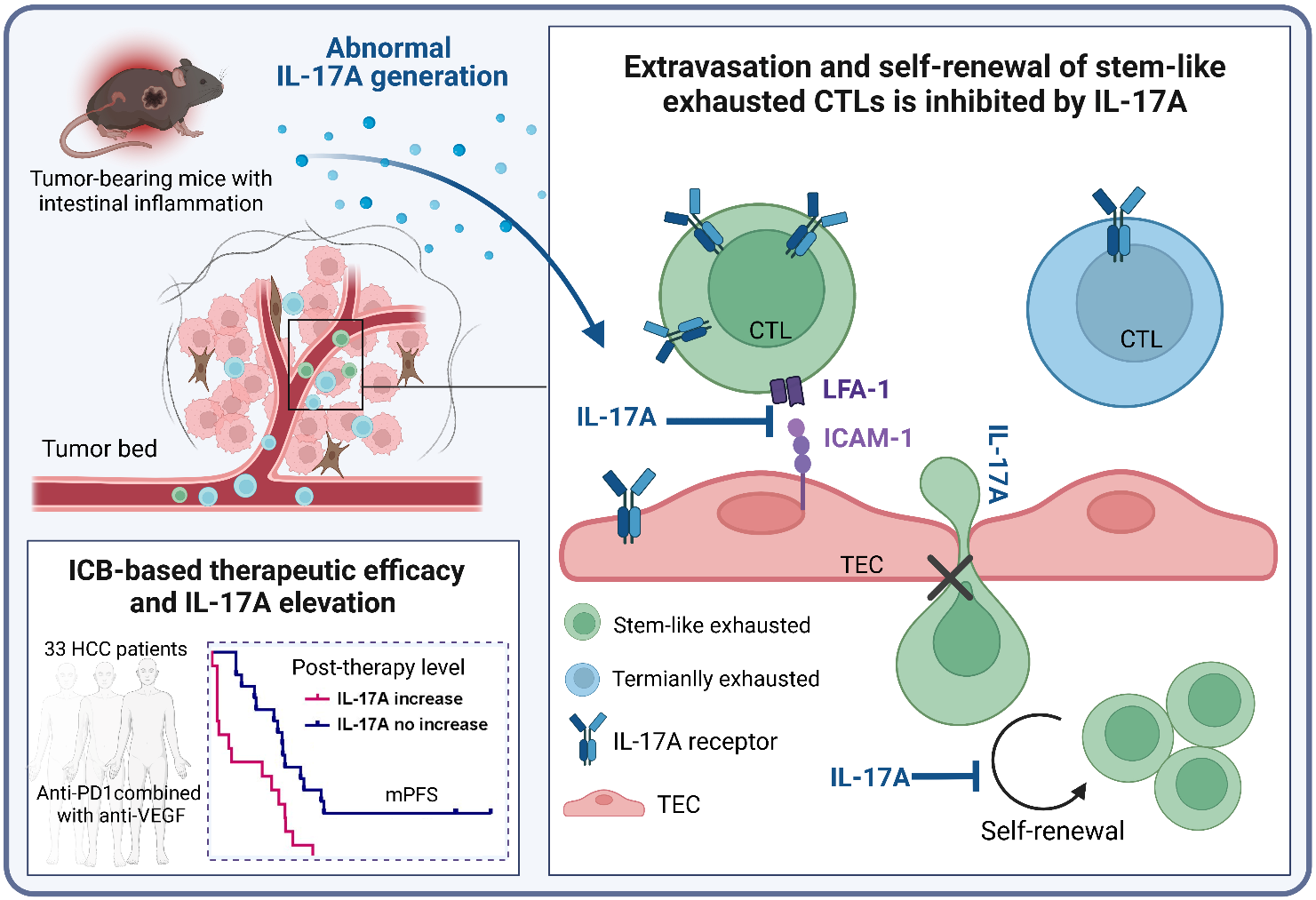


**Fig. S10.** **Graphic abstract**

The stem-like exhausted CTLs were recognized to express higher levels of IL-17A receptors, susceptible to IL-17A stimuation. Abbormal generation of IL-17A due to acute colitis diminished the interaction of stem-like exhausted CTLs with tumor vascular endothelium, restraining the cell extravasation, and inhibited the cell self-renewal in tumor beds. The graphic abstract was created with BioRender.com.
