# Supplementary material for: Abnormal generation of IL-17A represses tumor infiltration of stem-like exhausted CD8+ T cells to demote the antitumor immunity
Source: BMC Med. 2023 Aug 21;21:315. doi: 10.1186/s12916-023-03026-y (PMC10441727; doi:10.1186/s12916-023-03026-y)
Supplement: Supplementary file 3 — Additional file 3. The original immunoblot images of Fig. 5E. [file 12916_2023_3026_MOESM3_ESM.docx]

**Additional File for Review**

**BMED-D-23-01728R1**

**Title:** Abnormal generation of IL-17A represses tumor infiltration of stem-like exhausted CD8^+^ T cells to demote the antitumor immunity

**The original immunoblot images of Fig. 5E.**


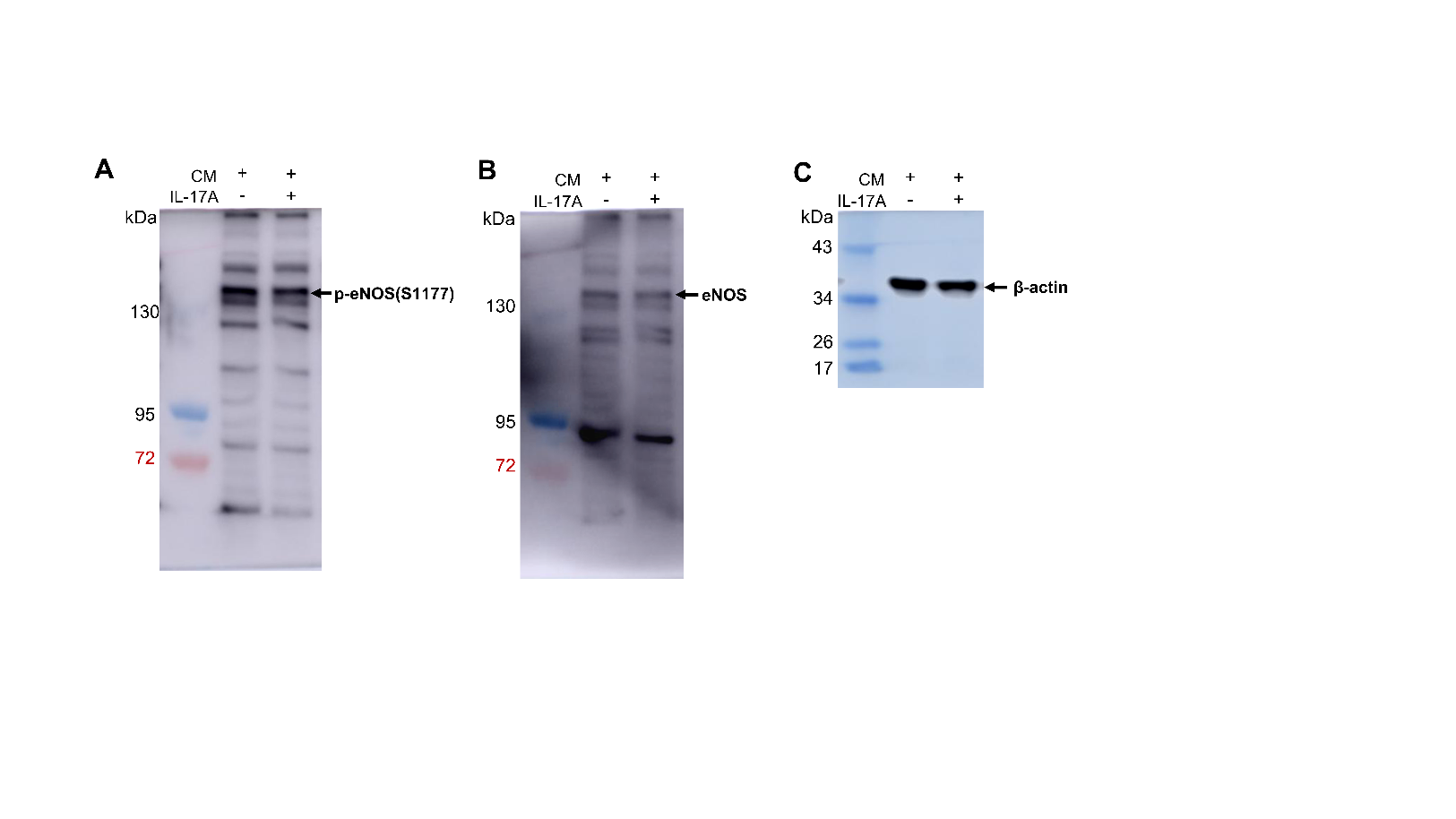


Detection of phosphorylated eNOS at Ser1177 and eNOS in differently treated C166 cells, β-actin as loading control. The images were captured using Amersham Imager 600 (GE Healthcare, MA, USA).

Figure 5E in the main body of the manuscript shows the original immunoblot images of phosphorylated eNOS at Ser1177(A), eNOS(B) and β-actin(C) (as indicated by arrows).
